# Toward Machine Learning Electrospray Ionization Sensitivity Prediction for Semiquantitative Lipidomics in Stem Cells

**DOI:** 10.1021/acs.jcim.4c02040

**Published:** 2025-02-05

**Authors:** Alexandria Van Grouw, Markace A. Rainey, Olivia K. Reid, Molly M. Ogle, Samuel G. Moore, Johnna S. Temenoff, Facundo M. Fernández

**Affiliations:** † School of Chemistry and Biochemistry, Georgia Institute of Technology, 901 Atlanta Drive, Atlanta, Georgia 30332, USA; ‡ The Wallace H. Coulter Department of Biomedical Engineering, Georgia Institute of Technology and Emory University, 313 Ferst Drive NW, Atlanta, Georgia 30332, USA; § Systems Mass Spectrometry Core, Parker H. Petit Institute for Bioengineering and Bioscience, Georgia Institute of Technology, 315 Ferst Drive NW, Atlanta, Georgia Samuel, USA

## Abstract

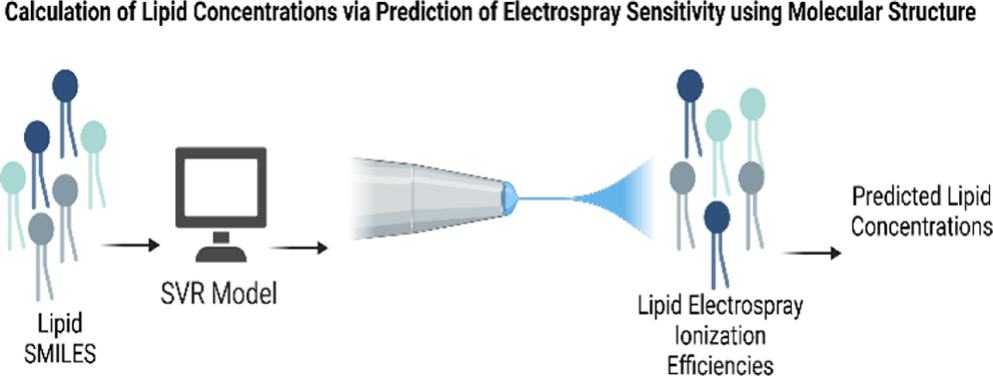

Specificity, sensitivity, and high metabolite coverage make mass spectrometry (MS) one of the most valuable tools in metabolomics and lipidomics. However, translation of metabolomics MS methods to multiyear studies conducted across multiple batches is limited by variability in electrospray ionization response, making batch-to-batch comparisons challenging. This limitation creates an artificial divide between nontargeted discovery work that is broad in scope but limited in terms of absolute quantitation ability and targeted work that is highly accurate but limited in scope due to the need for matched isotopically labeled standards. These issues are often observed in stem cell studies using metabolomic and lipidomic MS approaches, where patient recruitment can be a years-long process and samples become available in discrete batches every few months. To bridge this gap, we developed a machine learning model that predicts electrospray ionization sensitivity for lipid classes that have shown correlation with stem cell potency. Molecular descriptors derived from these lipids’ chemical structures are used as model input to predict electrospray response, enabling quantitation by MS with moderate accuracy (semiquantitation). Model performance was evaluated via internal and external validation using cultured cells from various stem cell donors, achieving global percent errors of 40% and 20% for positive and negative electrospray ion modes, respectively. Although this accuracy is typically insufficient for traditional targeted lipidomics experiments, it is sufficient for semiquantitative estimation of lipid marker concentrations across batches without the need for specific chemical standards that many times are unavailable. Furthermore, the precision for model-predicted concentrations was 16.9% for the positive mode and 7.5% for the negative mode, indicating promise for data harmonization across batches. The set of molecular descriptors used by the models described here was able to yield higher accuracy than those previously published in the literature, showing high promise toward semiquantitative lipidomics.

## 1 Introduction

Cell-based therapies hold great promise for the treatment of incurable diseases^1−3^ through cell replacement or release of trophic factors. Such therapies can be manufactured from a number of cell types, including primary, progenitor, or stem cells.^1^ The inherent complexity associated with cellular processes grants these therapies their unique potential but also makes consistent manufacturing at specific quality and safety levels a significant challenge.^4^ Mesenchymal stromal cells (MSCs) used in immunotherapy applications, for example, have been known to suffer from donor-to-donor variability and intracolony heterogeneity.^5^ In this context, discovery and quantitation of biochemical critical quality attributes (CQAs) indicative of relative potency and mechanisms of action are critical for standardizing treatments and ensuring the rigor of clinical trials.^5,6^

Metabolomics and lipidomics are emerging as sources of CQAs highly predictive of cell therapy properties, including potency.^7−11^ These ‘omic approaches produce metabolite abundance profiles that are the closest to the cellular phenotype at any given time point. Metabolomics/lipidomics experiments typically rely on liquid chromatography-mass spectrometry (LC-MS) conducted in a nontargeted fashion to discover CQAs,^12^ producing relative quantitation data within a given batch of experiments. The transition from nontargeted data sets to targeted quantitative CQA assays is often difficult to achieve,^13,14^ limiting the comparison of CQA abundances across batches and cell types. For example, a recent MSC metabolomics study on three cell lines identified four ether-linked phosphatidylcholine (O-PC) lipid CQA candidates that correlated with inhibited T-cell proliferation during coculture.^9^ However, a follow-up expanded study on ten cell lines showed that these markers were not as strongly correlated, while a small panel of different phospholipids were better predictors.^11^

While the standard practices of quantitative (targeted) metabolomics and lipidomics are well-established, the practices of the corresponding nontargeted workflows have not yet been standardized, emphasizing the need for data harmonization.^13,14^ Nontargeted CQA discovery work is broad in scope but limited in terms of quantitative ability, while targeted work is highly accurate but limited in scope due to the need for expensive stable isotope-labeled standards.^13,14^ Semiquantitative metabolomics and lipidomics methods leverage some of the advantages of targeted and nontargeted approaches by utilizing surrogate standards to obtain quantitative information for hundreds of analytes in a single experiment, but at the cost of some accuracy.^14,15^ Drotlef et al., for example, developed a lipid class-specific calibration workflow with stable isotope-labeled standards achieving acceptable accuracy levels, but the approach required the use of a reference material to calculate the necessary response factors.^16^ The use of such surrogate standards alone, however, cannot correct common effects observed in the LC-MS electrospray ionization (ESI) ion source, such as differential nonlinear responses, variations in matrix effects, or differences in analyte ionization efficiencies within a lipid class.^17,18^

Machine learning (ML) has played a role in the merging of nontargeted and targeted metabolomics/lipidomics LC-MS workflows.^19^ Both random forest (RF) and artificial neural networks (ANN) have been used to predict ESI sensitivity based on analyte properties such as LC-MS chromatographic retention time, capillary electrophoresis effective charge, and even fragmentation information from incomplete structural assignments.^20−22^ These studies were able to achieve regression models with *R*^2^ values above 0.8, but in both cases, the models performed poorly on independent data sets. Approaches based on molecular descriptors have also been used to predict relative ionization sensitivity using support vector regression (SVR), RF, ANN, and other ML techniques, but the work was limited to a very narrow data set comprising only carboxylic acids in negative ion mode.^23^ The most successful molecular descriptor-based studies for the prediction of ionization efficiency have achieved excellent *R*^2^ results on training and test sets but had errors as high as ∼200% when predicting the concentration of analytes in real samples.^24−26^ These studies were conducted without accounting for matrix effects and included very large training sets that span many chemical classes. Here, we present the first results of a semiquantitative LC-MS lipidomics approach that includes stable isotope surrogate internal standards, matrix matching, and ML relative ionization sensitivity prediction in order to account for all major sources of inaccuracy encountered in MSC lipid quantitation experiments.

## 2 Experimental Procedures

### 2.1 Preparation of Standard Mixtures

Avanti Polar Lipids UltimateSPLASH ONE mix was diluted with isopropanol to produce six calibrant stock solutions (UCal mixes) with 1:500 (Calibrant 1), 1:200 (Calibrant 2), 1:67 (Calibrant 3), 1:33 (Calibrant 4), 1:17 (Calibrant 5), and 1:10 (Calibrant 6) dilution factors. Avanti Polar Lipids SPLASH LIPIDOMIX was diluted 1:113 in isopropanol to create a High Splash Lipidomix stock and 1:581 for a Low Splash Lipidomix stock.

### 2.2 Sample Preparation and Extraction

**Figure 1:**
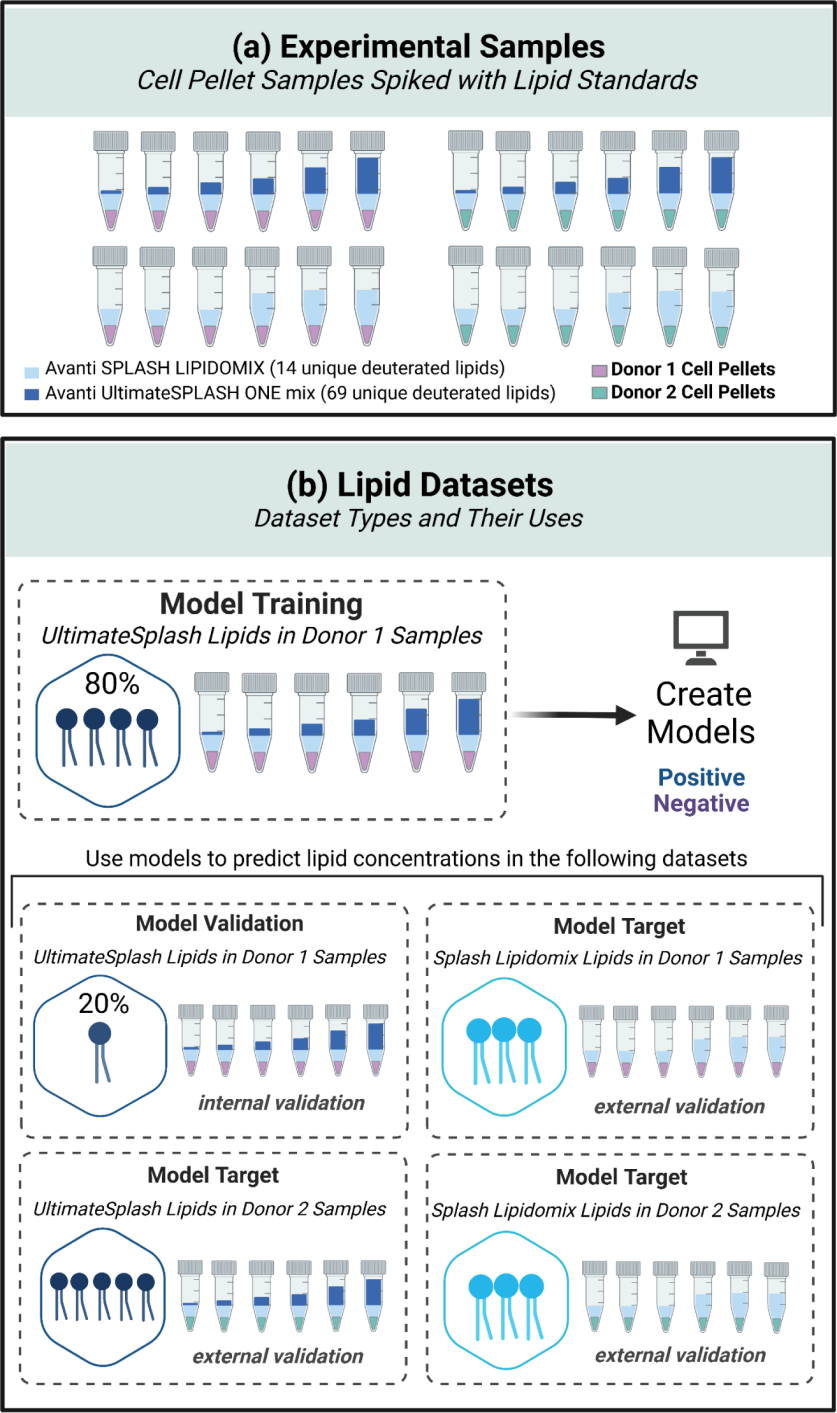
Collection of training and evaluation data sets for machine learning models. Two lipid standard mixes, the Avanti UltimateSPLASH ONE and Avanti SPLASH LIPIDOMIX, were spiked according to the (a) scheme into cell pellet samples composed of MSCs from two donors. From these samples, several lipid (b) data sets were created for model training, testing, and target evaluation. UI27RMEWO3 Created in BioRender. Fernandez, F. (2025).

Commercially available human bone marrow MSC samples from two donors (#182, #310 RoosterBio, Inc.) were thawed and allowed to recover in the culture for 4 days in a standard cell culture incubator at 37 °C with 5% CO_2_. The culture media were composed of low glucose Dulbecco’s Minimal Essential Medium (DMEM, Gibco) containing pyruvate, l-glutamine, penicillin-streptomycin, and 10% fetal bovine serum (FBS). Cells were washed three times in phosphate-buffered saline (PBS) and harvested by scraping with a sterile cell scraper. Cells were counted by a hemocytometer and aliquoted into 12 replicates from each donor at approximately 200,000 cells per sample. Cells were pelleted in 250 μL of methanol and 155 mM ammonium acetate. The supernatant was removed from all samples prior to the addition of extraction solvent and lipid standard mixes. Isopropanol, UCal standards specific for each calibration level, and/or Splash Lipidomix standards were added to all cellular samples and blanks for extraction according to Table S1. A representative scheme of sample components is shown in Figure 1a. Following the addition of the appropriate extraction solvent spiked with lipid standards, 50 μL of glass beads (400–600 μm) was added. Samples were then bead homogenized on Tissuelyzer II at 30 Hz for 5 min, followed by centrifugation at 21,000 g for 5 min. The supernatant was transferred to new microcentrifuge vials. The original vial still containing the beads was washed with an additional 200 μL of 2-propanol, vortexed, and centrifuged, and the supernatant was transferred again to the new vial. Samples were then concentrated using vacuum centrifugation and reconstituted in 75 μL of isopropanol. Samples were then sonicated to ensure full reconstitution and centrifuged again. Five μL of each sample was removed to form a pooled QC. The remaining volume was transferred to LC-MS vials for analysis.

### 2.3 UHPLC-MS/MS Analysis

The lipidomics LC-MS workflow was carried out on a Thermo Orbitrap Exploris 240 (Thermo Fisher Scientific) system. Reverse phase LC was performed using an Accucore C30 column (2.1 mm × 150 mm, 2.6 μm particle size) on a Vanquish (Thermo Fisher Scientific) liquid chromatograph (Table S2). Full-scan data were collected at a mass resolution of 120,000 (fwhm) in Orbitrap. Data-dependent acquisition (DDA) was employed to yield fragmentation information on endogenous and spiked lipids in sample extracts. Collision energies of 15%, 30%, and 50% were chosen for the HCD MS^2^ experiments. LC-MS data were aligned and integrated, including blank removal, with Compound Discoverer 3.3 (Thermo Fisher Scientific). Drift correction was also performed in Compound Discoverer using responses from pooled QC samples that bracket sets of 20 individual samples. All lipid standards were annotated using exact mass matches.

### 2.4 Description of Machine Learning (ML) Data Sets

All (*m*/*z*, time) LC-MS features were exported from Compound Discoverer and separated into three lists: annotated endogenous lipid features, UltimateSplash lipid features, and Splash Lipidomix features. These peak areas automatically include all adducts that Compound Discoverer identified for that peak. A list of included adducts for all standards (and their relative abundances) can be found in Supporting Information. Responses (peak areas) for UltimateSplash lipids in donor 1 samples were used for model creation and internal validation. All other lipid data sets described in Figure 1b formed various lipid target sets for model external validation. Recovery of lipid standards was determined by spiking equivalent concentrations of the mixes into isopropanol and comparing peak areas between matrix and nonmatrix samples (Table S3).

### 2.5 Calculation of Experimental Sensitivity Values

**Figure 2:**
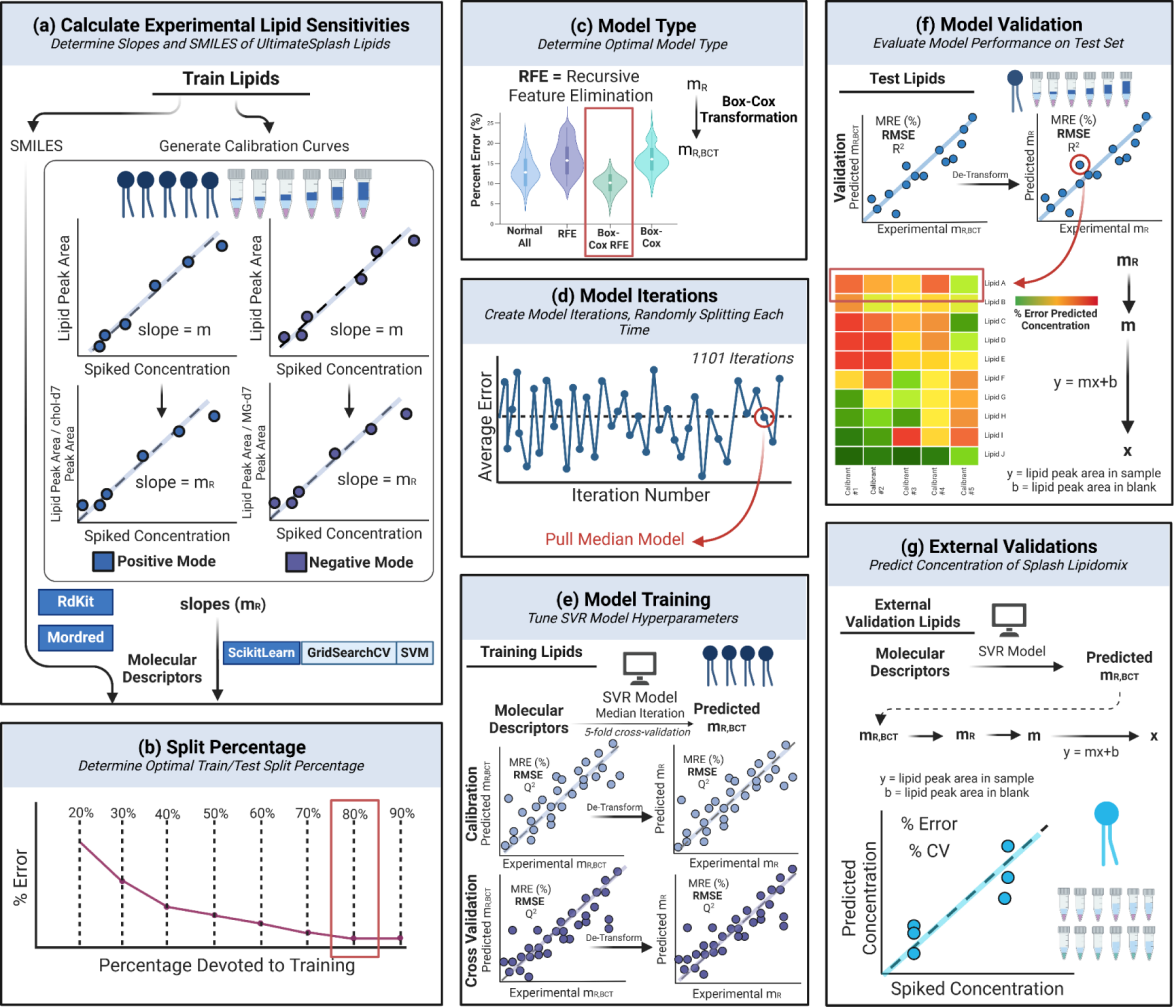
Model development workflow depicting (a) generation of lipid calibration curves for determination of experimental sensitivity values (*m*_R_), (b) identifying optimal train/test split percentage, (c) optimization of data transformation and feature selection, d) generation of 1,101 model iterations to select median error model, (e) model training with 5-fold cross validation, (f) model validation on test set lipids, and (g) external validation on separate lipids in separate samples. Full workflow results in two final models: one for each ionization mode, positive and negative. NA27RMF9PW Created in BioRender. Fernandez, F. (2025).

Peak areas for the UltimateSplash lipids in the six donor 1 calibrant samples were used to create external calibration curves based on their spiked concentrations. The slope (*m*) from these curves was calculated for all lipids observed in both positive and negative ion modes. Some lipids were not detected in both modes, and some lipids were removed because their calibration curves did not meet the linearity criterion (*R*^2^ < 0.8). For some lipids, the sixth and highest calibrator was outside the linearity range, so the sixth calibration level was removed for all lipids. A full list of lipids included in the final models can be found in Table S4a (positive ion mode) and Table S4b (negative ion mode). To control for injection-to-injection response differences, calibration curves were relativized to the cholesterol-d7 response (positive ion mode) and monoacylglycerol-d7 (negative ion mode), according to the workflow shown in Figure 2a. The resulting relativized slopes (*m*_R_) were used as the ground truth for support vector regression (SVR) model training.

### 2.6 Lipid Concentration Prediction Pipeline

A total of 61 and 50 lipid *m*_*R*_ values measured in positive and negative ion modes were used to create support vector machine (SVM) model sets. These model sets were first created by varying the percentages of lipids devoted to training and model testing (Figure 2b). Once an optimal training percentage of 80% was chosen, models of four different types were generated. Two of these models utilized recursive feature elimination (RFE) to remove nonuseful molecular descriptors, and two model types used a Box-Cox transformation to normalize the distribution of *m*_*R*_ values (Figure 2c). From the performance of these model types, it was determined that both the RFE and Box-Cox aided in model accuracy. Due to the relatively small lipid data set (UltimateSplash mix containing 69 unique lipids), there was concern that individual lipid allocation to train or test would have a significant impact on model performance. In order to mitigate testing effects, 1,101 model iterations were performed where the selection of the internal validation set was randomly generated in each iteration (Figure 2d). Following the creation of 1,101 models for each ionization mode, the median model based on the absolute error in the predicted lipid *m*_*R*_ was selected as the final model (Figure 2e).

### 2.7 Validation Procedures

Model internal validation was performed on the withheld 20% of UltimateSplash standards in donor 1 samples (Figure 2f). Predicted concentrations were determined by converting predicted *m*_R_ values to concentrations following eq 1, where *y* is the peak area response in the sample and *b* is the peak area in the blank.
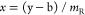
1

As an additional external validation, Splash Lipidomix standard concentrations were predicted in separate samples (Figure 2g).

### 2.8 Single-Point Calibration

Single-point calibrations were performed to compare against SVR model results by calculating the response ratio for the UltimateSplash component with the highest structural similarity to that of the Splash Lipidomix analyte. Structural similarity was defined as having the same headgroup, the closest hydrocarbon chain length and the closest number of double bonds. A table indicating which UltimateSplash component was used in each case is included (Table S5). The response factor was then applied to the Splash Lipidomix analyte to estimate its concentration.^27^ An example calculation is provided in Figure S1a.

### 2.9 Multipoint Calibration

A multipoint calibration approach using surrogate standards was also performed as a comparison to SVR model results. For this method, UltimateSplash components from a particular lipid class were used to create a calibration curve composed of different individual lipid members of a single class. The slope of that curve was then used to quantify the Splash Lipidomix analyte of the same class. An example calculation is provided in Figure S1b.

### 2.10 Machine Learning and Package Dependencies

All regression models were built in Python (v3.11.5) using Jupyter Notebook (v6.5.4) with IPykernel (v6.25.0). Plotting and visualization were performed using Matplotlib (v3.7.2), Plotly (v5.18.0), and Seaborn (v0.12.2) libraries. Molecular representations were generated via RDKit (v2023.09.5), and the corresponding molecular descriptors were calculated by using Mordred (v1.2.0). General data manipulation and analysis were facilitated by NumPy (v1.24.3), Pandas (v2.1.4), and SciPy (v1.11.1). Machine learning models were built and refined using the scikit-learn (v1.3.0) library. Data export and handling of Excel files were managed using Pandas and Openpyxl (v3.0.10). Optionally, the PubChemPy (v1.0.4) library could be used for retrieving compound data from PubChem if a CID list is provided, although SMILES strings were used directly in this study.

## 3 Results

### 3.1 Experimentally Observed Sensitivities by Lipid Class

**Figure 3:**
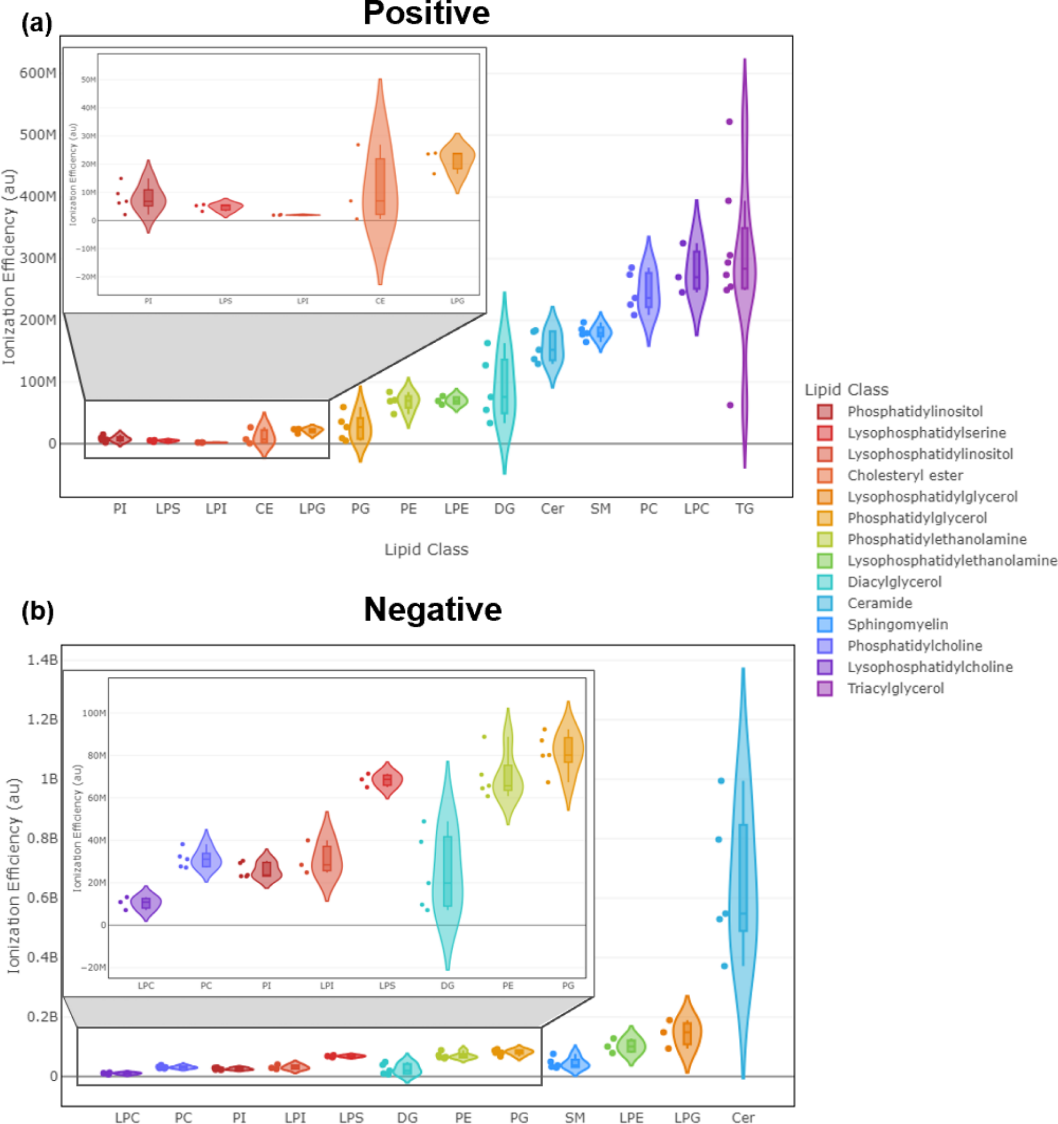
Electrospray ionization efficiency violin plots of observed lipid classes in (a) positive ion mode and (b) negative ion mode. The plot displays the ionization efficiencies of the lipid components of the Avanti Ultimate SplashOne mix spiked into donor 1 and donor 2 MSC samples. For each individual lipid, the ionization efficiencies measured were averaged across donor 1 and donor 2 samples. Ionization efficiency was defined as the slope of the concentration–response curve (*m*) using non-normalized peak areas.

The relative electrospray ionization efficiency of a given chemical species is largely controlled by its structure, other sample or solvent components, and the specific experimental conditions used, such as flow rate, solvent type, applied electric field strength, etc. For lipids, the molecular properties of the headgroup are important determinants of ionization efficiency, with the lipid tail chain lengths also having a marked impact. Lipids can also undergo in-source fragmentation with ESI, which will result in signal loss. In order to ensure ionization efficiency measurements were not significantly biased due to in-source fragmentation signal loss, common in-source fragments were identified and tabulated (Supporting Information). The observed range of experimental ionization efficiencies (*m*, Figure 2a) for the various tested lipid classes (Figure 3) was as expected. Additionally, some lipid classes showed a large within-class ionization efficiency range (Figure 3). Of particular note were the triacylglycerols (TG) in positive ion mode and ceramides (Cer) in negative ion mode. This variability highlighted the marked effects of tail lengths on ionization efficiencies and the challenges involved in using surrogate standards for quantification. Attempting to quantify analytes from such highly variable lipid classes using a surrogate standard could introduce inaccuracies in the predicted concentrations.

### 3.2 Prediction of Lipid Sensitivities

To evaluate the efficacy of our SVM regression workflow, a learning curve was generated by altering the percentage of UltimateSplash donor 1 lipids that were allocated to the training and internal validation steps (Figure S2). As expected, a median absolute percent error (MdAPE) plateau starting at train/test split ratios of 70:30 to 80:20 was observed for both positive and negative ion mode SVR models. Box-Cox transformation and RFE variable selection had minor but measurable effects at a split ratio of 80:20 when examining the MdAPE of the predicted relative sensitivities (*m*_R_). However, Box-Cox transformation clearly aided in model accuracy when examining the mean percent error, rather than MdAPE, for predicted lipid *m*_R_ by helping to prevent a few cases of high errors (Figure S3). RFE improved MdAPE, and for this reason, it was applied together with the Box-Cox transformation to the final workflow.

### 3.3 SVR Model Interpretability

The most commonly utilized molecular descriptors in all SVM model iterations were GAT descriptors (Figure S4). These are autocorrelation descriptors that represent the spatial distribution of atom or bond properties, explaining their correlation with the electrospray ionization efficiency. GAT descriptors examine how a molecular property is distributed across the molecular topography.^28^ For the positive ion mode in particular, GAT descriptors with a lag of 6 (GATS6xx), referring to the topological distance or path length, were important for SVM models.^28^ GAT autocorrelation descriptors used in SVR models were those weighted by properties such as electronegativity (e.g., *GATS6se*, *GATS6pe*, and *GATS6are*), valence electrons (*GATS1dv*), or intrinsic state (*GATS 3s*).

### 3.4 SVR Model Performance

**Figure 4:**
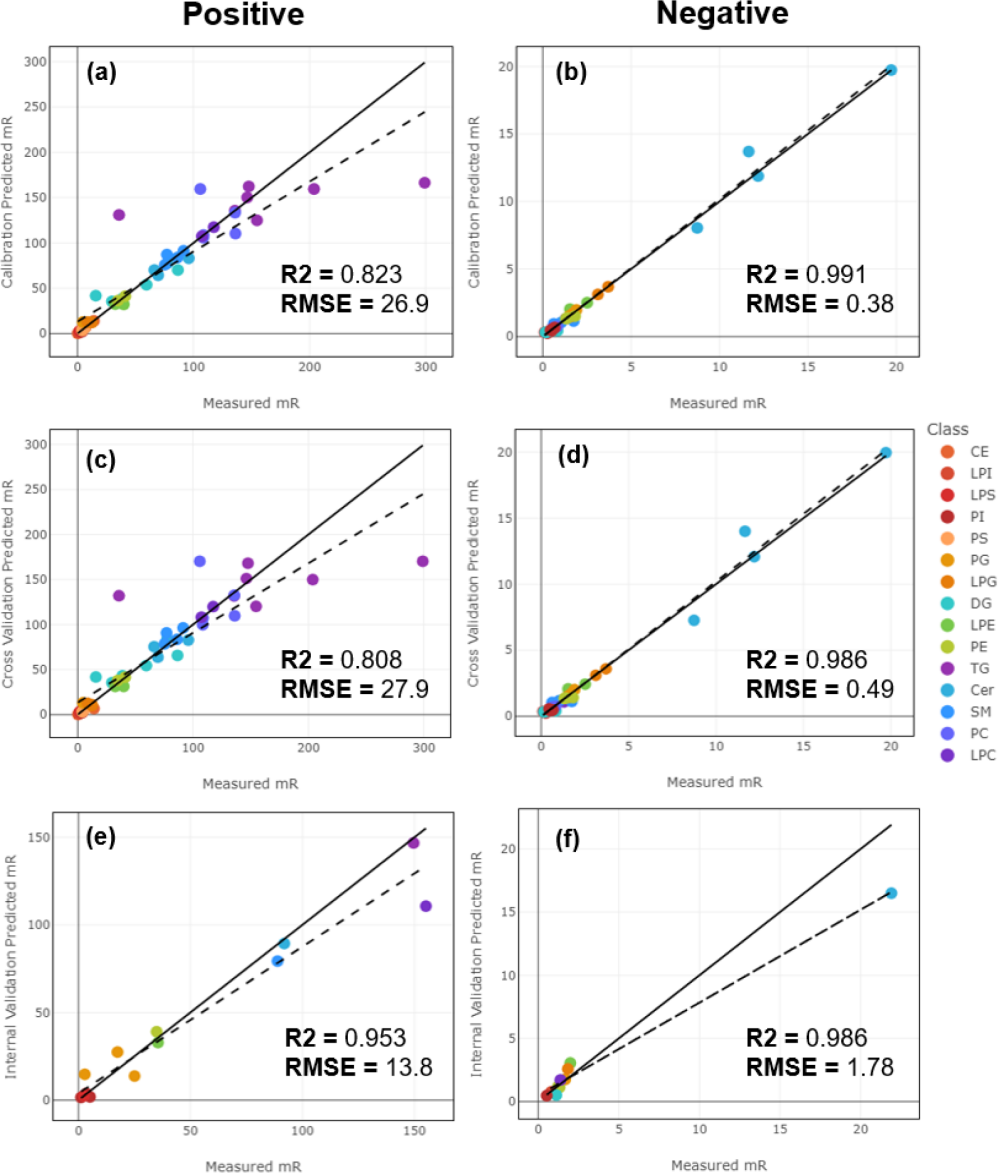
Model calibration and internal validation performance. Plots depicting measured relative slopes (*m*_R_) for UltimateSplash components in donor 1 samples against their predicted relative slopes for (a) positive ion mode model calibration, (b) negative ion mode calibration, (c) positive model cross-validation, (d) negative mode cross-validation, (e) positive mode internal validation, and (f) negative mode internal validation. For positive and negative ion mode model iterations, the median was chosen for display. All models utilized Box-Cox transformation and recursive feature elimination (RFE). Dashed lines indicate the regression fit line, and solid lines indicate a *x* = *y* fit.

**Figure 5:**
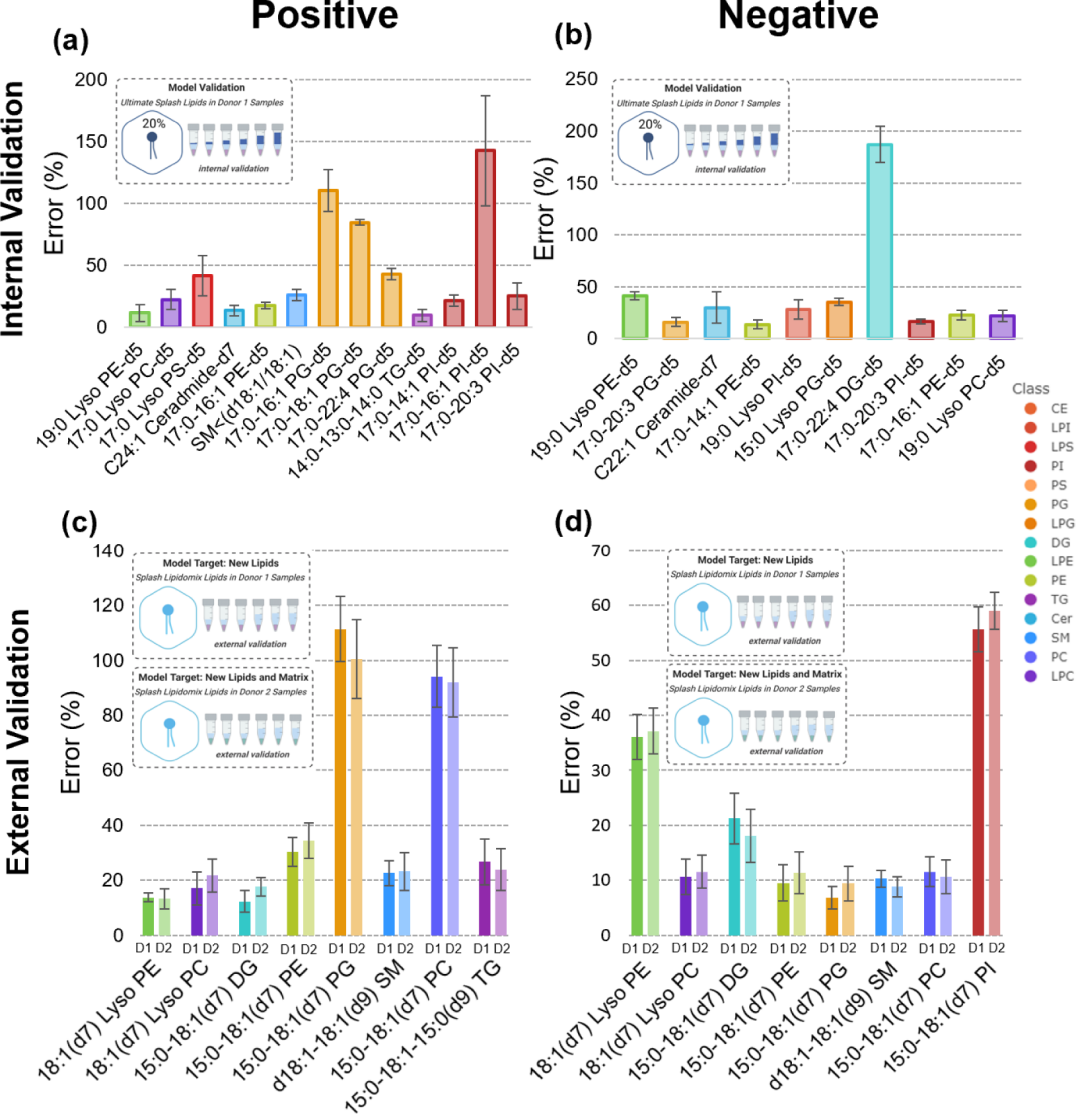
Errors for internal and external validation of positive and negative ion mode models. Average percent errors for (a) positive and (b) negative mode concentration predictions during internal validation using 20% of the analytes in UltimateSplash Mix. Errors were averaged at each calibration level. Average percent errors for (c) positive and (d) negative mode concentration predictions during external validation using Splash Lipidomix in donor 1 and donor 2 samples. Errors were averaged across three high samples and three low samples. Errors were calculated by comparing predicted concentrations to spiked concentrations. Note: ceramide names abbreviated for space; full names can be found in Table S3.

Both positive and negative mode models performed with high calibration accuracy (Figure 4a,b), as well as high performance in cross-validation (Figure 4c,d) with *R*-squared values of 0.808 for the positive model and 0.986 for the negative model. Following the assessment of model performance under calibration and cross-validation, the performance under internal validation conditions was evaluated. Ionization efficiencies for the 20% of left-out lipids in the UltimateSplash mix spiked into donor 1 samples were predicted (Figure 4e,f). The observed *R*-squared values were 0.953 and 0.986 for positive and negative ion modes, respectively, indicating the adequate predictive ability of the model on unseen data. The predicted *m*_R_ values for these internal validation standards were then used to calculate the concentrations of the matching lipids in each of the donor 1 samples in the internal validation set (Figure 5a,b). Concentration prediction errors were at or below 40%, with the exception of a few cases of higher errors, which included two phosphatidylglycerols (PGs) in positive mode, a phosphatidylinositol (PI) in positive mode, and a diacylglycerol (DG) in negative mode. For the PG and PI lipids, their errors are likely due to testing effects, as three out of five total class members were allocated to the internal validation set, leaving only two members in the training set. The concentration error for DG can be explained by the class’s large range of ionization efficiencies in negative mode. The global average error including these higher error cases was 43.6% for positive mode and 41.3% for negative mode. If these four lipid outliers are removed, the global average for positive ion mode was 22.9% and 25.1% in negative mode. Although errors in the 20–30% range would typically be unacceptable for strict quantitation work, they could still be acceptable for semiquantitative work in a nontargeted metabolomics setting where strict accuracy is not the ultimate goal of method development. The overall precision for internal calibration was determined by taking the mean of the coefficients of variation of each lipid determined from the predicted concentrations in the 5 samples. The precision was 22.6% for the positive mode and 15.7% for the negative mode. Errors for specific samples and lipids in the internal validation sets are provided in Figure S5 and S6.

### 3.5 Model Performance in External Validation Sets

Real-life quantitation using nontargeted metabolomics data sets would require that accuracy be maintained even if the ionization prediction model is applied to analytes not present in the training set. In addition, accuracy should be maintained across different sample types, ideally even across different matrices. In order to assess ionization prediction accuracy in these stringent conditions, external validation sets comprised of Splash Lipidomix lipids were also tested (Figure 5c,d). If outliers were removed, the global average errors for positive and negative ion modes were 21.4% and 15.2%, respectively. However, a few cases of higher errors were observed, specifically for PG (15:0–18:1(d7)) and PC (15:0–18:1(d7)) in positive mode and PI (15:0–18:1(d7)) in negative mode. The global average error including these higher error cases was 40.9% and 20.9% in positive and negative modes, respectively. The precision for the predicted concentrations was 16.9% for the positive mode and 7.5% for the negative mode. Errors for individual samples and lipid analytes in the external validation sets are detailed in Figures S7–10. Overall, these results indicated that predicting lipid concentrations in a variety of matrices with acceptable accuracy was possible for a number of different lipids as long as their lipid classes were well-represented in the training set. From these results, it was also apparent that matrix effects from individual cell donors were not significant enough to disrupt concentration prediction accuracy.

### 3.6 Outlier Analysis

A few of the lipids in the external validation set had high prediction errors compared with the average (Figure S11). In the case of PI (15:0–18:1(d7)) in negative mode (Figure S11a), the source of the observed high error is likely the product of the innate model accuracy. However, for the two Splash Lipidomix standards measured in positive mode, PG(15:0–18:1(d7)) and PC(15:0–18:1(d7)) (Figure S11b,c), the errors may have resulted from additional factors. Testing effects may have caused these high errors given the relatively few examples of PC and PG lipids in the data set (5 each). Analysis of Figure S11b,c shows that PG (15:0–18:1(d7)) and PC (15:0–18:1(d7)) had much higher than expected responses, which deviated from the expected trend line. Examination of the retention times of these outlier lipids did not show them outside the range of retention times of training lipids (Tables SI 4a and SI 4b). Errors may be introduced if the external validation lipid has a much higher (or lower) relative sensitivity than the same lipid class components in the training set. As discussed in Figure 3, lipid ionization efficiency showed a significantly large range. Another possible explanation for the observed errors is ionization enhancement or suppression due to coeluting compounds present in cell extracts that did not affect ionization of training lipids. The percent recovery for PC (15:0–18:1(d7)) is over 100%, which supports that this error was likely due to ionization enhancement in this case. Although spiking of deuterated standards into real biological samples should mitigate these effects due to matrix matching, it might not offer a universal mitigation of matrix effects in all possible scenarios, such as when there is coelution of highly ionizable lipids.

### 3.7 Comparison to Traditional Quantitation Approaches

**Figure 6:**
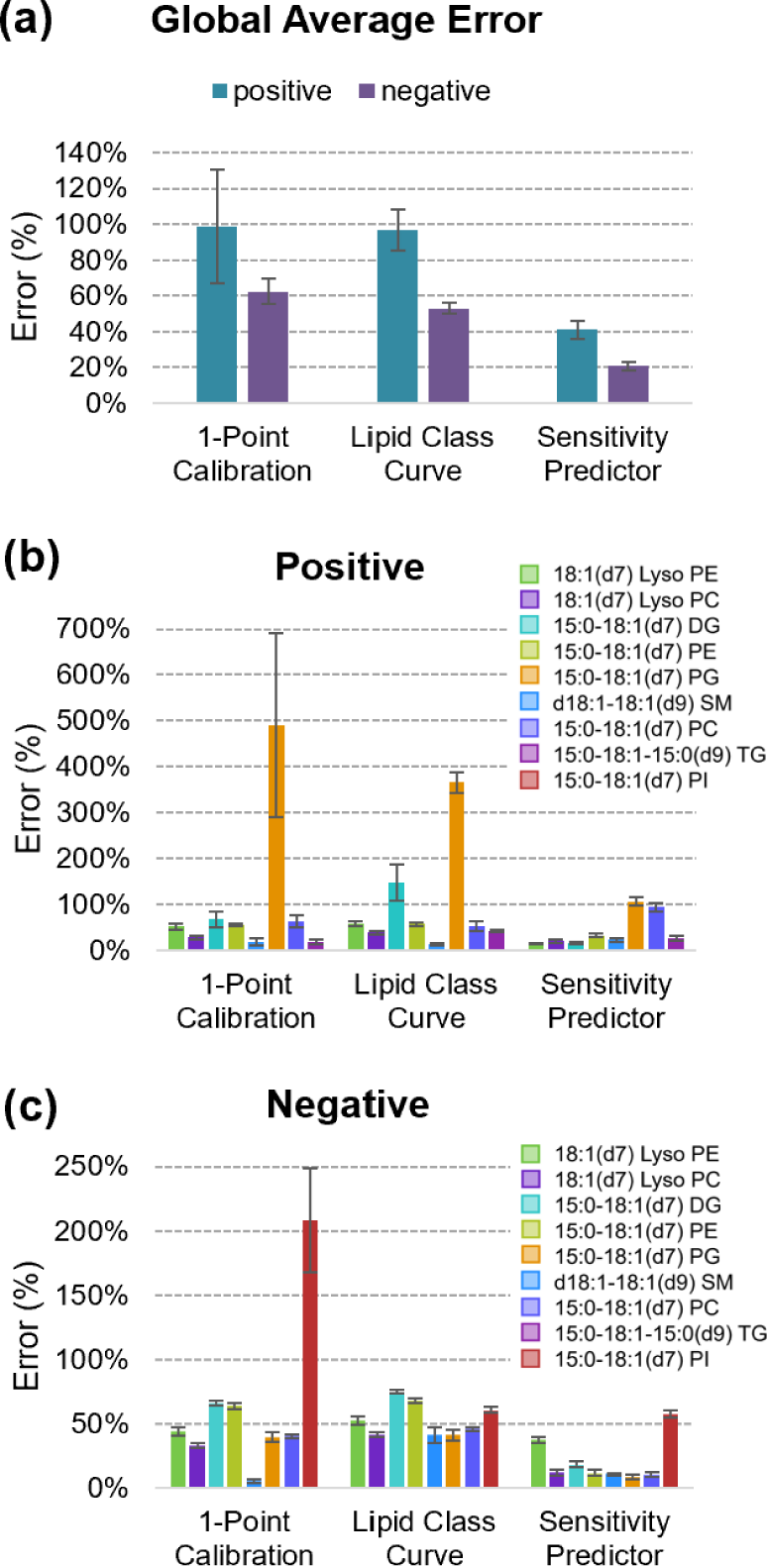
Model accuracy compared to traditional nontargeted quantitation approaches. The accuracies of predicted and calculated concentrations for Splash Lipidomix standards in donor 1 and donor 2 samples (averaged across 12 samples). Concentrations were determined using the prediction workflow, one-point calibration, and surrogate lipid class curve calibration and are displayed as (a) a global average for positive and negative modes, (b) averages by lipid component in positive mode, and (c) averages by lipid component in negative mode.

More often than not, quantitation in lipidomics is performed using simple calibration approaches such as 1-point calibration or establishing a least-squares linear regression for a specific lipid class. For these approaches, surrogate chemical standards are used to determine a response relationship that is applied to all lipid analytes of the same class, even if they are not perfectly matched to the standard. For comparison purposes, we applied two semiquantitative approaches described in Figure S1a. These methods resulted in concentration estimation errors higher than the ML-based approach developed in this work (Figure 6a). The approach with the highest mean error was the 1-point approach, with errors ranging from 100% to 60% in positive and negative ionization modes. The lipid class-specific calibration curves showed lower mean errors (50–90%), but still higher than the SVR model, which was between 20 and 40%. When the performance was evaluated on a lipid class basis for each ionization mode, the simpler quantitation methods were often sufficiently accurate but failed for PG (15:0–18:1(d7)) and DG (15:0–18:1(d7)) in positive mode, and PI (15:0–18:1(d7)) in negative mode. The SVR regression approach showed more accurate concentration estimates than one-point calibrations and lipid-class specific calibration curves in all examined cases.

### 3.8 Model Performance on Unseen Lipid Classes

Many lipids from bacteria and mammalian cells are modified from the lipid classes presented here or exist in different classes altogether. To examine how well the model can generalize to new lipid classes, all lyso lipids were removed from the training set and designated as a separate validation set. In this experiment, the model was trained on all lipids, excluding lyso lipids. The performance in this experiment indicates that the model does not easily predict unseen classes, as the *R*^2^ for model validations was −16.3 and −151.7 in positive and negative mode, respectively (Figure S12). This result is not surprising given the nature of machine learning. Without the necessary trends represented in the training set, extrapolation by the model is usually unsuccessful. Another way to examine robustness, however, is to examine how well the model performs within each class instead of how it performs out-of-class. For example, we may want to know whether it can extrapolate to quantify very large or very small lipids. The largest ultimate lipids (by mass) from each class were removed for the test set (Figure S13), and the smallest lipids (by mass) from each class were removed (Figure S13). *R*-squared measurements for these validation sets were all between 0.75 and 1, with greater accuracy in the large lipid validation sets, whose *R*-squared measurements were both between 0.95 and 1. This indicates that the model can extrapolate for lipid chain lengths beyond what it has seen in training.

## 4 Conclusions

We demonstrate a powerful approach for semiquantitation in nontargeted lipidomics workflows that utilizes machine learning prediction of ionization efficiency for concentration estimation. Although this approach requires the use of lipid standards for matrix matching, it enables the quantitation of thousands of lipids from only 69 standards and enables the quantitation of some lipids for which standards do not exist. The prediction of concentration is more accurate than simple nontargeted calibration approaches and can be achieved with high precision, indicating promise for use in studies for which conducting LC-MS lipidomics in multiple sample batches is necessary. The model presented here was created with cellular samples, but this approach could be adopted in many types of sample matrices and is customizable to other instrumentation. Without the need to reoptimize the modeling approach, creation of project/instrument-specific models is simple. The length of time that a single model could hold for continued batches in a project is yet to be determined, but here we demonstrate the general feasibility of this approach for semiquantitation. This method could be used to determine relevant biological lipid concentrations to inform the development of targeted assays directed from nontargeted data, aiding biomarker discovery work. Data harmonization and lipidomics data set comparison remain the most promising future application of this work, as meta-analysis of several cellular lipidomics studies remains a challenge in the field that accurate semiquantitation could help address.

## Data Availability

The full Jupyter notebook for sensitivity prediction is publicly available at https://github.com/facundof2016/Sensitivity_Predictor.git. All raw data and input files including lipid SMILES are also present in the repository.

## Supporting Information Available

Sample preparation and analysis details, example calculations, learning curves, model type comparison, most-selected Mordred descriptors, heatmaps of validation set prediction error by sample, and outlier analysis (PDF)
Adduct and in-source fragment ratio table (XLSX)

## References

[ref1] Buzhor E.; Leshansky L.; Blumenthal J.; Barash H.; Warshawsky D.; Mazor Y.; Shtrichman R. Cell-Based Therapy Approaches: The Hope for Incurable Diseases. Regen. Med. 2014, 9 (5), 649–672. 10.2217/rme.14.35. 25372080

[ref2] Song N.; Scholtemeijer M.; Shah K. Mesenchymal Stem Cell Immunomodulation: Mechanisms and Therapeutic Potential. Trends Pharmacol. Sci. 2020, 41 (9), 653–664. 10.1016/j.tips.2020.06.009. 32709406 PMC7751844

[ref3] Yamanaka S. Pluripotent Stem Cell-Based Cell Therapy—Promise and Challenges. Cell Stem Cell 2020, 27 (4), 523–531. 10.1016/j.stem.2020.09.014. 33007237

[ref4] Brachtl G.; Poupardin R.; Hochmann S.; Raninger A.; Jürchott K.; Streitz M.; Schlickeiser S.; Oeller M.; Wolf M.; Schallmoser K.; Volk H.-D.; Geissler S.; Strunk D. Batch Effects during Human Bone Marrow Stromal Cell Propagation Prevail Donor Variation and Culture Duration: Impact on Genotype, Phenotype and Function. Cells 2022, 11 (6), 946 10.3390/cells11060946. 35326396 PMC8946746

[ref5] Galipeau J.; Sensébé L. Mesenchymal Stromal Cells: Clinical Challenges and Therapeutic Opportunities. Cell Stem Cell 2018, 22 (6), 824–833. 10.1016/j.stem.2018.05.004. 29859173 PMC6434696

[ref6] Wuchter P.; Bieback K.; Schrezenmeier H.; Bornhäuser M.; Müller L. P.; Bönig H.; Wagner W.; Meisel R.; Pavel P.; Tonn T.; Lang P.; Müller I.; Renner M.; Malcherek G.; Saffrich R.; Buss E. C.; Horn P.; Rojewski M.; Schmitt A.; Ho A. D.; Sanzenbacher R.; Schmitt M. Standardization of Good Manufacturing Practice–Compliant Production of Bone Marrow–Derived Human Mesenchymal Stromal Cells for Immunotherapeutic Applications. Cytotherapy 2015, 17 (2), 128–139. 10.1016/j.jcyt.2014.04.002. 24856898

[ref7] Culberson A. L.; Bowles-Welch A. C.; Wang B.; Kottke P. A.; Jimenez A. C.; Roy K.; Fedorov A. G. Early Detection and Metabolic Pathway Identification of T Cell Activation by In-Process Intracellular Mass Spectrometry. Cytotherapy 2023, 25 (9), 1006–1015. 10.1016/j.jcyt.2023.03.010. 37061898 PMC10524195

[ref8] Jimenez A. C.; Heist C. A.; Navaei M.; Yeago C.; Roy K. Longitudinal Two-Dimensional Gas Chromatography Mass Spectrometry as a Non-Destructive at-Line Monitoring Tool during Cell Manufacturing Identifies Volatile Features Correlative to Cell Product Quality. Cytotherapy 2022, 24 (11), 1136–1147. 10.1016/j.jcyt.2022.06.001. 35882596

[ref9] Maughon T. S.; Shen X.; Huang D.; Michael A. O. A.; Shockey W. A.; Andrews S. H.; McRae J. M.; Platt M. O.; Fernández F. M.; Edison A. S.; Stice S. L.; Marklein R. A. Metabolomics and Cytokine Profiling of Mesenchymal Stromal Cells Identify Markers Predictive of T-Cell Suppression. Cytotherapy 2022, 24 (2), 137–148. 10.1016/j.jcyt.2021.08.002. 34696960

[ref10] Nikitina A. A.; Van Grouw A.; Roysam T.; Huang D.; Fernández F. M.; Kemp M. L. Mass Spectrometry Imaging Reveals Early Metabolic Priming of Cell Lineage in Differentiating Human-Induced Pluripotent Stem Cells. Anal. Chem. 2023, 95 (11), 4880–4888. 10.1021/acs.analchem.2c04416. 36898041 PMC10034746

[ref11] Van Grouw A.; Colonna M. B.; Maughon T. S.; Shen X.; Larey A. M.; Moore S. G.; Yeago C.; Fernández F. M.; Edison A. S.; Stice S. L.; Bowles-Welch A. C.; Marklein R. A. Development of a Robust Consensus Modeling Approach for Identifying Cellular and Media Metabolites Predictive of Mesenchymal Stromal Cell Potency. Stem Cells 2023, 41 (8), 792–808. 10.1093/stmcls/sxad039. 37279550 PMC10427967

[ref12] Domenick T. M.; Gill E. L.; Vedam-Mai V.; Yost R. A. Mass Spectrometry-Based Cellular Metabolomics: Current Approaches, Applications, and Future Directions. Anal. Chem. 2021, 93 (1), 546–566. 10.1021/acs.analchem.0c04363. 33146525

[ref13] Rampler E.; Abiead Y. E.; Schoeny H.; Rusz M.; Hildebrand F.; Fitz V.; Koellensperger G. Recurrent Topics in Mass Spectrometry-Based Metabolomics and Lipidomics—Standardization, Coverage, and Throughput. Anal. Chem. 2021, 93 (1), 519–545. 10.1021/acs.analchem.0c04698. 33249827 PMC7807424

[ref14] Cajka T.; Fiehn O. Toward Merging Untargeted and Targeted Methods in Mass Spectrometry-Based Metabolomics and Lipidomics. Anal. Chem. 2016, 88 (1), 524–545. 10.1021/acs.analchem.5b04491. 26637011

[ref15] Liu K. H.; Nellis M.; Uppal K.; Ma C.; Tran V.; Liang Y.; Walker D. I.; Jones D. P. Reference Standardization for Quantification and Harmonization of Large-Scale Metabolomics. Anal. Chem. 2020, 92 (13), 8836–8844. 10.1021/acs.analchem.0c00338. 32490663 PMC7887762

[ref16] Drotleff B.; Illison J.; Schlotterbeck J.; Lukowski R.; Lämmerhofer M. Comprehensive Lipidomics of Mouse Plasma Using Class-Specific Surrogate Calibrants and SWATH Acquisition for Large-Scale Lipid Quantification in Untargeted Analysis. Anal. Chim. Acta 2019, 1086, 90–102. 10.1016/j.aca.2019.08.030. 31561798

[ref17] Konermann L.; Ahadi E.; Rodriguez A. D.; Vahidi S. Unraveling the Mechanism of Electrospray Ionization. Anal. Chem. 2013, 85 (1), 2–9. 10.1021/ac302789c. 23134552

[ref18] Yu H.; Huan T. Patterned Signal Ratio Biases in Mass Spectrometry-Based Quantitative Metabolomics. Anal. Chem. 2021, 93 (4), 2254–2262. 10.1021/acs.analchem.0c04113. 33400486

[ref19] Groff L. C.; Grossman J. N.; Kruve A.; Minucci J. M.; Lowe C. N.; McCord J. P.; Kapraun D. F.; Phillips K. A.; Purucker S. T.; Chao A.; Ring C. L.; Williams A. J.; Sobus J. R. Uncertainty Estimation Strategies for Quantitative Non-Targeted Analysis. Anal. Bioanal. Chem. 2022, 414 (17), 4919–4933. 10.1007/s00216-022-04118-z. 35699740 PMC9465984

[ref20] Chalcraft K. R.; Lee R.; Mills C.; Britz-McKibbin P. Virtual Quantification of Metabolites by Capillary Electrophoresis-Electrospray Ionization-Mass Spectrometry: Predicting Ionization Efficiency Without Chemical Standards. Anal. Chem. 2009, 81 (7), 2506–2515. 10.1021/ac802272u. 19275147

[ref21] Palm E.; Kruve A. Machine Learning for Absolute Quantification of Unidentified Compounds in Non-Targeted LC/HRMS. Molecules 2022, 27 (3), 1013 10.3390/molecules27031013. 35164283 PMC8840743

[ref22] Sepman H.; Malm L.; Peets P.; MacLeod M.; Martin J.; Breitholtz M.; Kruve A. Bypassing the Identification: MS2Quant for Concentration Estimations of Chemicals Detected with Nontarget LC-HRMS from MS ^2^ Data. Anal. Chem. 2023, 95 (33), 12329–12338. 10.1021/acs.analchem.3c01744. 37548594 PMC10448440

[ref23] Mayhew A. W.; Topping D. O.; Hamilton J. F. New Approach Combining Molecular Fingerprints and Machine Learning to Estimate Relative Ionization Efficiency in Electrospray Ionization. ACS Omega 2020, 5 (16), 9510–9516. 10.1021/acsomega.0c00732. 32363303 PMC7191837

[ref24] Abrahamsson D.; Park J.-S.; Singh R. R.; Sirota M.; Woodruff T. J. Applications of Machine Learning to In Silico Quantification of Chemicals without Analytical Standards. J. Chem. Inf. Model. 2020, 60 (6), 2718–2727. 10.1021/acs.jcim.9b01096. 32379974 PMC7328371

[ref25] Liigand J.; Wang T.; Kellogg J.; Smedsgaard J.; Cech N.; Kruve A. Quantification for Non-Targeted LC/MS Screening without Standard Substances. Sci. Rep. 2020, 10 (1), 5808 10.1038/s41598-020-62573-z. 32242073 PMC7118164

[ref26] Bieber S.; Letzel T.; Kruve A. Electrospray Ionization Efficiency Predictions and Analytical Standard Free Quantification for SFC/ESI/HRMS. J. Am. Soc. Mass Spectrom. 2023, 34 (7), 1511–1518. 10.1021/jasms.3c00156. 37358930 PMC10326909

[ref27] Medina J.; Borreggine R.; Teav T.; Gao L.; Ji S.; Carrard J.; Jones C.; Blomberg N.; Jech M.; Atkins A.; Martins C.; Schmidt-Trucksass A.; Giera M.; Cazenave-Gassiot A.; Gallart-Ayala H.; Ivanisevic J. Omic-Scale High-Throughput Quantitative LC–MS/MS Approach for Circulatory Lipid Phenotyping in Clinical Research. Anal. Chem. 2023, 95 (6), 3168–3179. 10.1021/acs.analchem.2c02598. 36716250

[ref28] Hollas B. An Analysis of the Autocorrelation Descriptor for Molecules. J. Math. Chem. 2003, 33, 91–101. 10.1023/A:1023247831238.

