# Supplementary material for: Toward Machine Learning Electrospray Ionization Sensitivity Prediction for Semiquantitative Lipidomics in Stem Cells
Source: J Chem Inf Model. 2025 Feb 5;65(4):1826–36. doi: 10.1021/acs.jcim.4c02040 (PMC11863365; doi:10.1021/acs.jcim.4c02040)
Supplement: Supplementary file 1 — ci4c02040_si_001.pdf [file ci4c02040_si_001.pdf]

Towards Machine Learning Electrospray Ionization Sensitivity Prediction for Semi-quantitative Lipidomics in Stem Cells.

Alexandria Van Grouw[1], Markace A. Rainey[1], Olivia K. Reid[1], Molly M. Ogle[2], Samuel G. Moore[3], Johnna S. Temenoff[2], Facundo M. Fernández[1,3]

(1) School of Chemistry and Biochemistry, Georgia Institute of Technology, 901 Atlanta Drive, Atlanta, Georgia, USA

(2) The Wallace H. Coulter Department of Biomedical Engineering, Georgia Institute of Technology and Emory University, 313 Ferst Drive NW, Atlanta, Georgia, USA

(3) Systems Mass Spectrometry Core, Parker H. Petit Institute for Bioengineering and Bioscience, 315 Ferst Drive NW, Georgia Institute of Technology, Atlanta, Georgia, USA

Table of Contents

Table S1. List of all samples and lipid standard spike volumes……………………….S2
Table S2. UHPLC chromatographic gradient………………………………………….S3
Table S3 Lipid Standard Recovery…………………………… ……………………..S4
Table S4a. List of all lipid standards and the corresponding dataset (Train/Internal Validation/External Validation) for positive mode……………………………………S4
Table S4b. List of all lipid standards and the corresponding dataset (Train/Internal Validation/External Validation) for negative mode……………………………………S5
Figure S1. Example calculations for other semi-quantitative approaches……………S6
Table S5. List of corresponding Ultimate Splash standards used for quantitation of Splash Lipidomix standards in one-point calibration…………………………………S7
Figure S2. Learning curves……………………………………………………….....S8
Figure S3. Assessment of model performance for various approaches to data transformation and variable selection…………………………………………….....S9
Figure S4. Commonly selected variables in model iterations…………………………S10
Figure S5-S10 Validation Heatmaps…………………………………………...S11-S16
Figure S11. Outlier Analysis……………………………………………………......S17
Figure S12. Model Performance on Unseen Lipid Classes…………………………...S18
Figure S13. Model Performance on Unseen Lipid Masses…………………………...S19

						Vol (µL)						
**Ultimate Splash Spiked Samples - Donors 1 and 2**												
	**D1 -Cal 1**	**D1 -Cal 2**	**D1-Cal 3**	**D1-Cal 4**	**D1-Cal 5**	**D1-Cal 6**	**D2-Cal1**	**D2-Cal 2**	**D2- Cal 3**	**D2 - Cal 4**	**D2 - Cal 5**	**D2 - Cal 6**
LOW Splash Lipidomix	500	500	500	500	500	500	500	500	500	500	500	500
Designated UCal Mix	100	100	100	100	100	100	100	100	100	100	100	100
**Splash Lipidomix Spiked Samples - Donors 1 and 2**												
	**D1-High 1**	**D1-High 2**	**D1-High 3**	**D1-Low 1**	**D1-Low 2**	**D1-Low 3**	**D2-High 1**	**D2-High 2**	**D2-High 3**	**D2-Low 1**	**D2-Low 2**	**D2-Low 3**
HIGH Splash Lipidomix	500	500	500	0	0	0	500	500	500	0	0	0
LOW Splash Lipidomix	0	0	0	500	500	500	0	0	0	500	500	500
Isopropanol (IPA)	100	100	100	100	100	100	100	100	100	100	100	100
Blank	600uL IPA											

Table S1. List of all samples and lipid standard spike volumes. Specified volumes of Designated UCal Mix and/or Splash Lipidomix and/or isopropanol (IPA) were added to cell pellets from Donor 1 (D1) and Donor 2 (D2) prior to applying the extraction protocol.

Reverse Phase Chromatography	
0-1 min	80% Mobile Phase A
1-5 min	40% Mobile Phase A
5-5.5 min	30% Mobile Phase A
5.5-8 min	15% Mobile Phase A
8-8.2 min	10% Mobile Phase A
8.2-10.5 min	0% Mobile Phase A
10.7-12 min	80% Mobile Phase A

Table S2. UHPLC chromatographic gradient. Mobile phase A used for positive ion mode was 40:60 water: acetonitrile with 10 mM ammonium formate and 0.1% formic acid. Mobile phase B was 10:90 acetonitrile:isopropyl alcohol, with 10 mM ammonium formate and 0.1% formic acid. For negative ionization mode, the mobile phases were 40:60 water:acetonitrile with 10 mM ammonium acetate (mobile phase A) and 10:90 acetonitrile:isopropyl alcohol, with 10 mM ammonium acetate (mobile phase B). The flow rate was set at 0.40 mL min^{-1}. The column temperature was set to 50 °C, and the injection volume was 2 µL.

POSITIVE		
Lipid Standard Name	Mean Recovery (%)	Recovery Standard Deviation (%)
15:0 Lyso PI-d5	9.52%	2.82%
17:0 Lyso PI-d5	9.41%	2.45%
19:0 Lyso PI-d5	8.39%	2.31%
15:0 Lyso PS-d5	26.22%	6.68%
17:0 Lyso PS-d5	28.31%	9.35%
19:0 Lyso PS-d5	18.67%	8.53%
15:0 Lyso PG-d5	74.41%	9.63%
17:0 Lyso PG-d5	70.77%	10.85%
19:0 Lyso PG-d5	67.93%	10.07%
15:0 Lyso PE-d5	39.01%	5.13%
17:0 Lyso PE-d5	33.50%	4.09%
19:0 Lyso PE-d5	31.53%	4.10%
18:1(d7) Lyso PE	29.08%	5.14%
15:0 Lyso PC-d5	39.93%	6.30%
17:0 Lyso PC-d5	36.72%	6.91%
19:0 Lyso PC-d5	32.16%	5.48%
18:1(d7) Lyso PC	30.40%	7.01%
17:0-14:1 PI-d5	23.45%	5.62%
17:0-16:1 PI-d5	21.66%	4.67%
17:0-18:1 PI-d5	24.03%	6.30%
17:0-20:3 PI-d5	20.34%	4.10%
17:0-22:4 PI-d5	25.67%	6.14%
15:0-18:1(d7) PI	29.79%	9.70%
16:1 SM (d18:1/16:1)-d9	44.08%	8.32%
18:1 SM (d18:1/18:1)-d9	52.97%	7.18%
20:1 SM (d18:1/20:1)-d9	60.05%	7.13%
22:1 SM (d18:1/22:1)-d9	54.35%	3.52%
24:1 SM (d18:1/24:1)-d9	47.65%	3.60%
d18:1-18:1(d9) SM	59.64%	6.51%
17:0-14:1 PS-d5	47.78%	12.26%
17:0-16:1 PS-d5	73.51%	16.03%
17:0-20:3 PS-d5	52.72%	14.53%
17:0-22:4 PS-d5	removed	removed
17:0-14:1 PG-d5	33.84%	4.06%
17:0-16:1 PG-d5	33.62%	4.19%
17:0-18:1 PG-d5	23.54%	6.61%
17:0-20:3 PG-d5	removed	removed
17:0-22:4 PG-d5	36.07%	3.22%
15:0-18:1(d7) PG	33.20%	4.87%
17:0-14:1 PC-d5	49.59%	5.83%
17:0-16:1 PC-d5	68.84%	35.14%
17:0-18:1 PC-d5	46.06%	4.95%
17:0-20:3 PC-d5	66.43%	7.54%
17:0-22:4 PC-d5	52.28%	4.27%
15:0-18:1(d7) PC	158.44%	15.99%
17:0-14:1 PE-d5	34.05%	3.36%
17:0-16:1 PE-d5	31.68%	3.33%
17:0-18:1 PE-d5	36.85%	3.20%
17:0-20:3 PE-d5	removed	removed
17:0-22:4 PE-d5	36.44%	30.83%
15:0-18:1(d7) PE	31.44%	5.64%
C16:1 Ceramide-d7 (d18:1-d7/16:1)	35.24%	3.95%
C18:1 Ceramide-d7 (d18:1-d7/18:1)	37.55%	5.73%
C20:1 Ceramide-d7 (d18:1-d7/20:1)	45.78%	6.60%
C22:1 Ceramide-d7 (d18:1-d7/22:1)	37.43%	4.87%
C24:1 Ceramide-d7 (d18:1-d7/24:1)	34.27%	3.16%
16:1 cholesteryl-d7 ester	40.82%	6.69%
20:3 Cholesteryl-d7 ester	65.66%	24.10%
22:4 cholesteryl-d7 ester	54.35%	8.17%
	28.65%	6.18%
17:0-14:1 DG-d5	52.39%	7.29%
17:0-16:1 DG-d5	33.52%	4.71%
17:0-18:1 DG-d5	43.33%	4.11%
17:0-20:3 DG-d5	40.75%	3.11%
17:0-22:4 DG-d5	52.92%	10.73%
15:0-18:1(d7) DG	58.51%	7.00%
14:0-13:0-14:0 TG-d5	43.45%	5.00%
14:0-15:1-14:0 TG-d5	43.20%	5.11%
14:0-17:1-14:0 TG-d5	14.26%	3.51%
16:0-15:1-16:0 TG-d5	34.81%	4.51%
16:0-17:1-16:0 TG-d5	removed	removed
16:0-19:2-16:0 TG-d5	43.11%	9.18%
18:1-17:1-18:1 TG-d5	84.14%	15.21%
18:1-19:2-18:1 TG-d5	40.55%	13.66%
18:1-21:2-18:1 TG-d5	41.95%	5.47%
15:0-18:1(d7)-15:0 TG	58.91%	14.61%

NEGATIVE		
Lipid Standard Name	Mean Recovery (%)	Recovery Standard Deviation (%)
15:0 Lyso PI-d5	15.70%	4.71%
17:0 Lyso PI-d5	15.54%	4.72%
19:0 Lyso PI-d5	13.09%	4.52%
15:0 Lyso PS-d5	29.35%	23.87%
17:0 Lyso PS-d5	22.91%	18.92%
19:0 Lyso PS-d5	16.00%	13.70%
15:0 Lyso PG-d5	42.73%	6.26%
17:0 Lyso PG-d5	49.66%	8.65%
19:0 Lyso PG-d5	44.76%	9.52%
15:0 Lyso PE-d5	41.29%	7.11%
17:0 Lyso PE-d5	36.72%	7.14%
19:0 Lyso PE-d5	34.73%	6.02%
18:1(d7) Lyso PE	21.12%	5.57%
15:0 Lyso PC-d5	26.82%	21.63%
17:0 Lyso PC-d5	24.57%	20.16%
19:0 Lyso PC-d5	23.93%	19.51%
18:1(d7) Lyso PC	34.64%	10.46%
17:0-14:1 PI-d5	20.20%	4.65%
17:0-16:1 PI-d5	20.03%	4.55%
17:0-18:1 PI-d5	19.12%	5.01%
17:0-20:3 PI-d5	20.33%	4.59%
17:0-22:4 PI-d5	21.24%	4.84%
15:0-18:1(d7) PI	27.78%	10.28%
16:1 SM (d18:1/16:1)-d9	35.63%	5.13%
18:1 SM (d18:1/18:1)-d9	38.13%	8.59%
20:1 SM (d18:1/20:1)-d9	31.71%	5.89%
22:1 SM (d18:1/22:1)-d9	39.60%	5.50%
24:1 SM (d18:1/24:1)-d9	33.65%	5.68%
d18:1-18:1(d9) SM	69.64%	21.68%
17:0-14:1 PG-d5	36.65%	4.82%
17:0-16:1 PG-d5	44.61%	8.92%
17:0-18:1 PG-d5	50.34%	3.97%
17:0-20:3 PG-d5	43.66%	5.23%
17:0-22:4 PG-d5	54.88%	13.01%
15:0-18:1(d7) PG	63.76%	17.33%
17:0-14:1 PC-d5	33.75%	5.41%
17:0-16:1 PC-d5	31.71%	4.91%
17:0-18:1 PC-d5	37.00%	5.68%
17:0-20:3 PC-d5	29.83%	5.71%
17:0-22:4 PC-d5	35.63%	5.56%
15:0-18:1(d7) PC	45.21%	11.90%
17:0-14:1 PE-d5	36.24%	3.81%
17:0-16:1 PE-d5	37.80%	4.34%
17:0-18:1 PE-d5	39.35%	5.28%
17:0-20:3 PE-d5	39.53%	4.12%
17:0-22:4 PE-d5	40.33%	4.60%
15:0-18:1(d7) PE	34.46%	9.82%
C16:1 Ceramide-d7 (d18:1-d7/16:1)	70.13%	18.34%
C18:1 Ceramide-d7 (d18:1-d7/18:1)	83.42%	17.83%
C20:1 Ceramide-d7 (d18:1-d7/20:1)	67.66%	9.07%
C22:1 Ceramide-d7 (d18:1-d7/22:1)	101.33%	17.97%
C24:1 Ceramide-d7 (d18:1-d7/24:1)	99.41%	18.40%
17:0-14:1 DG-d5	42.47%	5.97%
17:0-16:1 DG-d5	39.48%	5.63%
17:0-18:1 DG-d5	36.15%	5.55%
17:0-20:3 DG-d5	41.33%	6.12%
17:0-22:4 DG-d5	36.69%	6.38%
15:0-18:1(d7) DG	54.86%	13.89%
18:1(d7) MG	48.03%	16.13%

Table S3. Lipid Standard Recovery. Recovery values were determined by comparing peak area responses for standards spiked in cellular samples with responses for standards spiked into isopropanol. Recoveries for all samples were averaged, and their standard deviation calculated.

POSITIVE Lipid Standard Name	Lipid Mix	Dataset	Primary Adduct	*m/z*	RT	Monoisotopic Mass	Mass Error (ppm)	Samples	Spike Concentration (µg/mL)
15:0 Lyso PI-d5	Ultimate	Train	[M+H-H2O]+	546.30829	1.555	563.31185	-0.486	Calibration Points 1-6	0.067, 0.167, 0.50, 1.0, 2.0, 3.333
17:0 Lyso PI-d5	Ultimate	Train	[M+H-H2O]+	574.33954	2.120	591.34315	-0.548	Calibration Points 1-6	0.133, 0.333, 1.0, 2.0, 4.0, 6.667
19:0 Lyso PI-d5	Ultimate	Train	[M+H]+	620.38158	2.359	619.37445	-0.241	Calibration Points 1-6	0.067, 0.167, 0.50, 1.0, 2.0, 3.333
15:0 Lyso PS-d5	Ultimate	Train	[M+H]+	489.29808	1.690	488.29051	0.609	Calibration Points 1-6	0.067, 0.167, 0.50, 1.0, 2.0, 3.333
17:0 Lyso PS-d5	Ultimate	Int. Val	[M+H]+	517.32932	2.165	516.32181	0.460	Calibration Points 1-6	0.133, 0.333, 1.0, 2.0, 4.0, 6.667
19:0 Lyso PS-d5	Ultimate	Train	[M+H]+	545.36078	2.488	544.35311	0.730	Calibration Points 1-6	0.067, 0.167, 0.50, 1.0, 2.0, 3.333
15:0 Lyso PG-d5	Ultimate	Train	[M+H]+	476.30271	1.704	475.29531	0.268	Calibration Points 1-6	0.067, 0.167, 0.50, 1.0, 2.0, 3.333
17:0 Lyso PG-d5	Ultimate	Train	[M+H]+	504.33419	2.137	503.32661	0.611	Calibration Points 1-6	0.133, 0.333, 1.0, 2.0, 4.0, 6.667
19:0 Lyso PG-d5	Ultimate	Train	[M+H]+	532.36555	2.428	531.35791	0.691	Calibration Points 1-6	0.067, 0.167, 0.50, 1.0, 2.0, 3.333
15:0 Lyso PE-d5	Ultimate	Train	[M+H]+	445.30822	1.843	444.30130	-0.801	Calibration Points 1-6	0.067, 0.167, 0.50, 1.0, 2.0, 3.333
17:0 Lyso PE-d5	Ultimate	Train	[M+H]+	473.33945	2.253	472.33260	-0.902	Calibration Points 1-6	0.133, 0.333, 1.0, 2.0, 4.0, 6.667
19:0 Lyso PE-d5	Ultimate	Int. Val	[M+H]+	501.37095	2.589	500.36390	-0.452	Calibration Points 1-6	0.067, 0.167, 0.50, 1.0, 2.0, 3.333
18:1(d7) Lyso PE	Lipidomix	Ext. Val.	[M+H]+	487.35210	2.206	486.34510	-0.567	LOW 1-3, HIGH 1-3	0.061, 0.336
15:0 Lyso PC-d5	Ultimate	Train	[M+H]+	487.35523	1.913	486.34820	-0.506	Calibration Points 1-6	0.067, 0.167, 0.50, 1.0, 2.0, 3.333
17:0 Lyso PC-d5	Ultimate	Int. Val	[M+H]+	515.38648	2.293	514.37950	-0.575	Calibration Points 1-6	0.133, 0.333, 1.0, 2.0, 4.0, 6.667
19:0 Lyso PC-d5	Ultimate	Train	[M+H]+	543.41788	2.639	542.41080	-0.361	Calibration Points 1-6	0.067, 0.167, 0.50, 1.0, 2.0, 3.333
18:1(d7) Lyso PC	Lipidomix	Ext. Val	[M+H]+	529.39902	2.114	528.39210	-0.674	LOW 1-3, HIGH 1-3	0.293, 1.504
17:0-14:1 PI-d5	Ultimate	Int. Val	[M+NH4]+	817.55951	3.773	799.52595	-0.333	Calibration Points 1-6	0.067, 0.167, 0.50, 1.0, 2.0, 3.333
17:0-16:1 PI-d5	Ultimate	Int. Val	[M+NH4]+	845.59054	4.416	827.55725	-0.648	Calibration Points 1-6	0.133, 0.333, 1.0, 2.0, 4.0, 6.667
17:0-18:1 PI-d5	Ultimate	Train	[M+NH4]+	873.62178	5.227	855.58855	-0.697	Calibration Points 1-6	0.20, 0.50, 1.50, 3.0, 6.0, 10.0
17:0-20:3 PI-d5	Ultimate	Int. Val	[M+NH4]+	897.62183	4.808	879.58855	-0.621	Calibration Points 1-6	0.133, 0.333, 1.0, 2.0, 4.0, 6.667
17:0-22:4 PI-d5	Ultimate	Train	[M+NH4]+	923.63745	5.095	905.60415	-0.581	Calibration Points 1-6	0.067, 0.167, 0.50, 1.0, 2.0, 3.333
15:0-18:1(d7) PI	Lipidomix	Ext. Val.	[M+NH4]+	847.60321	4.358	829.56980	-0.498	LOW 1-3, HIGH 1-3	0.104, 0.537
16:1 SM (d18:1/16:1)-d9	Ultimate	Train	[M+H]+	710.61555	3.936	709.60840	-0.178	Calibration Points 1-6	0.20, 0.50, 1.50, 3.0, 6.0, 10.0
18:1 SM (d18:1/18:1)-d9	Ultimate	Int. Val	[M+H]+	738.64674	4.694	737.63970	-0.320	Calibration Points 1-6	0.133, 0.333, 1.0, 2.0, 4.0, 6.667
20:1 SM (d18:1/20:1)-d9	Ultimate	Train	[M+H]+	766.67808	5.550	765.67100	-0.256	Calibration Points 1-6	0.067, 0.167, 0.50, 1.0, 2.0, 3.333
22:1 SM (d18:1/22:1)-d9	Ultimate	Train	[M+H]+	794.70950	6.447	793.70230	-0.096	Calibration Points 1-6	0.133, 0.333, 1.0, 2.0, 4.0, 6.667
24:1 SM (d18:1/24:1)-d9	Ultimate	Train	[M+H]+	822.74046	7.169	821.73360	-0.506	Calibration Points 1-6	0.20, 0.50, 1.50, 3.0, 6.0, 10.0
d18:1-18:1(d9) SM	Lipidomix	Ext. Val.	[M+H]+	738.64674	4.694	737.63970	-0.320	LOW 1-3, HIGH 1-3	0.355, 1.823
17:0-14:1 PS-d5	Ultimate	Train	[M+H]+	725.51219	3.936	724.50461	0.424	Calibration Points 1-6	0.067, 0.167, 0.50, 1.0, 2.0, 3.333
17:0-16:1 PS-d5	Ultimate	Train	[M+H]+	753.54347	4.642	752.53591	0.382	Calibration Points 1-6	0.133, 0.333, 1.0, 2.0, 4.0, 6.667
17:0-20:3 PS-d5	Ultimate	Train	[M+H]+	805.57455	5.120	804.56721	0.084	Calibration Points 1-6	0.133, 0.333, 1.0, 2.0, 4.0, 6.667
17:0-22:4 PS-d5	Ultimate	Removed	[M+H]+	831.59013	5.283	830.58281	0.057	Calibration Points 1-6	0.067, 0.167, 0.50, 1.0, 2.0, 3.333
17:0-14:1 PG-d5	Ultimate	Train	[M+NH4]+	729.54321	3.942	711.50931	-0.113	Calibration Points 1-6	0.067, 0.167, 0.50, 1.0, 2.0, 3.333
17:0-16:1 PG-d5	Ultimate	Int. Val	[M+NH4]+	757.57471	4.625	739.54061	0.379	Calibration Points 1-6	0.133, 0.333, 1.0, 2.0, 4.0, 6.667
17:0-18:1 PG-d5	Ultimate	Int. Val	[M+Na]+	790.56099	5.462	767.57191	-0.175	Calibration Points 1-6	0.20, 0.50, 1.50, 3.0, 6.0, 10.0
17:0-20:3 PG-d5	Ultimate	Removed	[M+NH4]+	809.60574	5.022	791.57191	0.013	Calibration Points 1-6	0.133, 0.333, 1.0, 2.0, 4.0, 6.667
17:0-22:4 PG-d5	Ultimate	Int. Val	[M+NH4]+	835.62146	5.276	817.58761	0.037	Calibration Points 1-6	0.067, 0.167, 0.50, 1.0, 2.0, 3.333
15:0-18:1(d7) PG	Lipidomix	Ext. Val.	[M+NH4]+	759.58726	4.560	741.55370	-0.355	LOW 1-3, HIGH 1-3	0.334, 1.717
17:0-14:1 PC-d5	Ultimate	Train	[M+H]+	723.56942	4.404	722.56220	-0.078	Calibration Points 1-6	0.133, 0.333, 1.0, 2.0, 4.0, 6.667
17:0-16:1 PC-d5	Ultimate	Train	[M+H]+	751.60090	5.167	750.59350	0.165	Calibration Points 1-6	0.267, 0.667, 2.0, 4.0, 8.0, 13.333
17:0-18:1 PC-d5	Ultimate	Train	[M+H]+	779.63229	6.120	778.62480	0.275	Calibration Points 1-6	0.40, 1.0, 3.0, 6.0, 12.0, 20.0
17:0-20:3 PC-d5	Ultimate	Train	[M+H]+	803.63183	5.618	802.62480	-0.306	Calibration Points 1-6	0.267, 0.667, 2.0, 4.0, 8.0, 13.333
17:0-22:4 PC-d5	Ultimate	Train	[M+H]+	829.64766	5.905	828.64050	-0.140	Calibration Points 1-6	0.133, 0.333, 1.0, 2.0, 4.0, 6.667
15:0-18:1(d7) PC	Lipidomix	Ext. Val.	[M+H]+	753.61321	5.132	752.60610	-0.221	LOW 1-3, HIGH 1-3	1.844, 9.481
17:0-14:1 PE-d5	Ultimate	Train	[M+H]+	681.52237	4.530	680.51530	-0.303	Calibration Points 1-6	0.067, 0.167, 0.50, 1.0, 2.0, 3.333
17:0-16:1 PE-d5	Ultimate	Int. Val	[M+H]+	709.55369	5.349	708.54660	-0.263	Calibration Points 1-6	0.133, 0.333, 1.0, 2.0, 4.0, 6.667
17:0-18:1 PE-d5	Ultimate	Train	[M+H]+	737.58523	6.263	736.57790	0.073	Calibration Points 1-6	0.20, 0.50, 1.50, 3.0, 6.0, 10.0
17:0-20:3 PE-d5	Ultimate	Removed	[M+H]+	761.58594	5.781	760.57790	1.004	Calibration Points 1-6	0.133, 0.333, 1.0, 2.0, 4.0, 6.667
17:0-22:4 PE-d5	Ultimate	Removed	[M+H]+	787.60100	6.057	786.59350	0.285	Calibration Points 1-6	0.067, 0.167, 0.50, 1.0, 2.0, 3.333
15:0-18:1(d7) PE	Lipidomix	Ext. Val	[M+H]+	711.56635	5.270	710.55910	-0.037	LOW 1-3, HIGH 1-3	0.065, 0.336
C16:1 Ceramide-d7 (d18:1-d7/16:1)	Ultimate	Train	[M+Na]+	565.52917	4.712	542.54040	-0.826	Calibration Points 1-6	0.20, 0.50, 1.50, 3.0, 6.0, 10.0
C18:1 Ceramide-d7 (d18:1-d7/18:1)	Ultimate	Train	[M+H]+	571.57857	5.623	570.57170	-0.712	Calibration Points 1-6	0.133, 0.333, 1.0, 2.0, 4.0, 6.667
C20:1 Ceramide-d7 (d18:1-d7/20:1)	Ultimate	Train	[M+Na]+	621.59186	6.464	598.60300	-0.598	Calibration Points 1-6	0.067, 0.167, 0.50, 1.0, 2.0, 3.333
C22:1 Ceramide-d7 (d18:1-d7/22:1)	Ultimate	Train	[M+H]+	627.64133	6.860	626.63430	-0.393	Calibration Points 1-6	0.133, 0.333, 1.0, 2.0, 4.0, 6.667
C24:1 Ceramide-d7 (d18:1-d7/24:1)	Ultimate	Int. Val	[M+Na]+	677.65448	7.172	654.66560	-0.516	Calibration Points 1-6	0.20, 0.50, 1.50, 3.0, 6.0, 10.0
16:1 cholesteryl-d7 ester	Ultimate	Train	[M+NH4]+	647.64652	9.723	629.61280	-0.159	Calibration Points 1-6	0.133, 0.333, 1.0, 2.0, 4.0, 6.667
20:3 Cholesteryl-d7 ester	Ultimate	Train	[M+NH4]+	699.67792	9.763	681.64412	-0.004	Calibration Points 1-6	0.133, 0.333, 1.0, 2.0, 4.0, 6.667
22:4 cholesteryl-d7 ester	Ultimate	Train	[M+NH4]+	725.69369	9.783	707.65983	0.092	Calibration Points 1-6	0.067, 0.167, 0.50, 1.0, 2.0, 3.333
17:0-14:1 DG-d5	Ultimate	Train	[M+Na]+	580.49573	5.998	557.50680	-0.517	Calibration Points 1-6	0.067, 0.167, 0.50, 1.0, 2.0, 3.333
17:0-16:1 DG-d5	Ultimate	Train	[M+Na]+	608.52716	6.665	585.53810	-0.270	Calibration Points 1-6	0.133, 0.333, 1.0, 2.0, 4.0, 6.667
17:0-18:1 DG-d5	Ultimate	Train	[M+NH4]+	631.60314	6.987	613.56940	-0.135	Calibration Points 1-6	0.20, 0.50, 1.50, 3.0, 6.0, 10.0
17:0-20:3 DG-d5	Ultimate	Train	[M+NH4]+	655.60321	6.812	637.56940	-0.020	Calibration Points 1-6	0.133, 0.333, 1.0, 2.0, 4.0, 6.667
17:0-22:4 DG-d5	Ultimate	Train	[M+NH4]+	681.61865	6.892	663.58500	-0.261	Calibration Points 1-6	0.067, 0.167, 0.50, 1.0, 2.0, 3.333
15:0-18:1(d7) DG	Lipidomix	Ext. Val	[M+Na]+	610.53955	6.633	587.55060	0.456	LOW 1-3, HIGH 1-3	0.108, 0.555
14:0-13:0-14:0 TG-d5	Ultimate	Int. Val	[M+NH4]+	731.69182	8.063	713.65820	-0.284	Calibration Points 1-6	0.067, 0.167, 0.50, 1.0, 2.0, 3.333
14:0-15:1-14:0 TG-d5	Ultimate	Train	[M+NH4]+	757.70756	8.074	739.67380	-0.085	Calibration Points 1-6	0.133, 0.333, 1.0, 2.0, 4.0, 6.667
14:0-17:1-14:0 TG-d5	Ultimate	Train	[M+Na]+	790.69368	8.062	767.70510	-0.813	Calibration Points 1-6	0.20, 0.50, 1.50, 3.0, 6.0, 10.0
16:0-15:1-16:0 TG-d5	Ultimate	Train	[M+NH4]+	813.77033	8.985	795.73640	0.134	Calibration Points 1-6	0.267, 0.667, 2.0, 4.0, 8.0, 13.333
16:0-17:1-16:0 TG-d5	Ultimate	Removed	[M+Na]+	846.75615	8.482	823.76770	-0.832	Calibration Points 1-6	0.333, 0.834, 2.50, 5.0, 10.0, 16.667
16:0-19:2-16:0 TG-d5	Ultimate	Train	[M+NH4]+	867.81696	9.404	849.78340	-0.309	Calibration Points 1-6	0.267, 0.667, 2.0, 4.0, 8.0, 13.333
18:1-17:1-18:1 TG-d5	Ultimate	Train	[M+NH4]+	893.83275	9.346	875.79900	-0.083	Calibration Points 1-6	0.20, 0.50, 1.50, 3.0, 6.0, 10.0
18:1-19:2-18:1 TG-d5	Ultimate	Train	[M+NH4]+	919.84793	9.382	901.81470	-0.658	Calibration Points 1-6	0.133, 0.333, 1.0, 2.0, 4.0, 6.667
18:1-21:2-18:1 TG-d5	Ultimate	Train	[M+NH4]+	947.87993	9.570	929.84600	0.115	Calibration Points 1-6	0.067, 0.167, 0.50, 1.0, 2.0, 3.333
15:0-18:1(d7)-15:0 TG	Lipidomix	Ext. Val.	[M+NH4]+	829.79817	9.179	811.76460	-0.312	LOW 1-3, HIGH 1-3	0.658, 3.381

Table S4a. List of all lipid standards and the corresponding dataset (Train/Internal Validation/External Validation), primary observed ion adduct, the corresponding *m/z*, retention time, monoisotopic mass, mass error, samples containing that specific standard, and the spike concentration for standards in positive ion mode.

NEGATIVE<br>Lipid Standard Name	Lipid Mix	Dataset	Primary Adduct	m/z	RT	Monoisotopic Mass	Mass Error (ppm)	Samples	Spike Concentration (µg/mL)
15:0 Lyso PI-d5	Ultimate	Train	[M-H]-	562.30440	1.933	563.31185	0.314	Calibration Points 1-6	0.067, 0.167, 0.50, 1.0, 2.0, 3.333
17:0 Lyso PI-d5	Ultimate	Train	[M-H]-	590.33575	2.411	591.34315	0.215	Calibration Points 1-6	0.133, 0.333, 1.0, 2.0, 4.0, 6.667
19:0 Lyso PI-d5	Ultimate	Test	[M-H]-	618.36700	2.732	619.37445	0.286	Calibration Points 1-6	0.067, 0.167, 0.50, 1.0, 2.0, 3.333
15:0 Lyso PS-d5	Ultimate	Train	[M-H]-	487.28358	1.969	488.29051	-0.715	Calibration Points 1-6	0.067, 0.167, 0.50, 1.0, 2.0, 3.333
17:0 Lyso PS-d5	Ultimate	Train	[M-H]-	515.31491	2.495	516.32181	-0.735	Calibration Points 1-6	0.133, 0.333, 1.0, 2.0, 4.0, 6.667
19:0 Lyso PS-d5	Ultimate	Train	[M-H]-	543.34625	2.918	544.35311	-0.770	Calibration Points 1-6	0.067, 0.167, 0.50, 1.0, 2.0, 3.333
15:0 Lyso PG-d5	Ultimate	Int. Val	[M-H]-	474.28825	1.955	475.29531	-0.461	Calibration Points 1-6	0.067, 0.167, 0.50, 1.0, 2.0, 3.333
17:0 Lyso PG-d5	Ultimate	Train	[M-H]-	502.31964	2.497	503.32661	-0.615	Calibration Points 1-6	0.133, 0.333, 1.0, 2.0, 4.0, 6.667
19:0 Lyso PG-d5	Ultimate	Train	[M-H]-	530.35099	2.778	531.35791	-0.676	Calibration Points 1-6	0.067, 0.167, 0.50, 1.0, 2.0, 3.333
15:0 Lyso PE-d5	Ultimate	Train	[M-H]-	443.29373	2.356	444.30130	0.662	Calibration Points 1-6	0.067, 0.167, 0.50, 1.0, 2.0, 3.333
17:0 Lyso PE-d5	Ultimate	Train	[M-H]-	471.32496	2.734	472.33260	0.771	Calibration Points 1-6	0.133, 0.333, 1.0, 2.0, 4.0, 6.667
19:0 Lyso PE-d5	Ultimate	Int. Val	[M-H]-	499.35635	3.069	500.36390	0.548	Calibration Points 1-6	0.067, 0.167, 0.50, 1.0, 2.0, 3.333
18:1(d7) Lyso PE	Lipidomix	Ext. Val	[M-H]-	485.33756	2.625	486.34510	0.543	LOW 1-3, HIGH 1-3	0.061, 0.336
15:0 Lyso PC-d5	Ultimate	Train	[M+Acetate-H]-	545.36195	2.213	486.34820	0.208	Calibration Points 1-6	0.067, 0.167, 0.50, 1.0, 2.0, 3.333
17:0 Lyso PC-d5	Ultimate	Train	[M+Acetate-H]-	573.39321	2.703	514.37950	0.274	Calibration Points 1-6	0.133, 0.333, 1.0, 2.0, 4.0, 6.667
19:0 Lyso PC-d5	Ultimate	Int. Val	[M+Acetate-H]-	601.42448	3.029	542.41080	0.315	Calibration Points 1-6	0.067, 0.167, 0.50, 1.0, 2.0, 3.333
18:1(d7) Lyso PC	Lipidomix	Ext. Val	[M+Acetate-H]-	587.40572	2.592	528.39210	0.437	LOW 1-3, HIGH 1-3	0.293, 1.504
17:0-14:1 PI-d5	Ultimate	Train	[M-H]-	798.51852	3.775	799.52595	0.196	Calibration Points 1-6	0.067, 0.167, 0.50, 1.0, 2.0, 3.333
17:0-16:1 PI-d5	Ultimate	Train	[M-H]-	826.54991	4.284	827.55725	0.081	Calibration Points 1-6	0.133, 0.333, 1.0, 2.0, 4.0, 6.667
17:0-18:1 PI-d5	Ultimate	Train	[M-H]-	854.58107	4.953	855.58855	0.242	Calibration Points 1-6	0.20, 0.50, 1.50, 3.0, 6.0, 10.0
17:0-20:3 PI-d5	Ultimate	Int. Val	[M-H]-	878.58116	4.593	879.58855	0.133	Calibration Points 1-6	0.133, 0.333, 1.0, 2.0, 4.0, 6.667
17:0-22:4 PI-d5	Ultimate	Train	[M-H]-	904.59679	4.798	905.60415	0.096	Calibration Points 1-6	0.067, 0.167, 0.50, 1.0, 2.0, 3.333
15:0-18:1(d7) PI	Lipidomix	Ext. Val	[M-H]-	828.56255	4.233	829.56980	-0.031	LOW 1-3, HIGH 1-3	0.104, 0.537
16:1 SM (d18:1/16:1)-d9	Ultimate	Train	[M+Acetate-H]-	768.62221	4.223	709.60840	0.058	Calibration Points 1-6	0.20, 0.50, 1.50, 3.0, 6.0, 10.0
18:1 SM (d18:1/18:1)-d9	Ultimate	Train	[M+Acetate-H]-	796.65345	4.917	737.63970	0.137	Calibration Points 1-6	0.133, 0.333, 1.0, 2.0, 4.0, 6.667
20:1 SM (d18:1/20:1)-d9	Ultimate	Train	[M+Acetate-H]-	824.68484	5.727	765.67100	0.014	Calibration Points 1-6	0.067, 0.167, 0.50, 1.0, 2.0, 3.333
22:1 SM (d18:1/22:1)-d9	Ultimate	Train	[M+Acetate-H]-	852.71609	6.626	793.70230	0.077	Calibration Points 1-6	0.133, 0.333, 1.0, 2.0, 4.0, 6.667
24:1 SM (d18:1/24:1)-d9	Ultimate	Train	[M+Acetate-H]-	880.74741	7.196	821.73360	0.050	Calibration Points 1-6	0.20, 0.50, 1.50, 3.0, 6.0, 10.0
d18:1-18:1(d9) SM	Lipidomix	Ext. Val	[M+Acetate-H]-	796.65345	4.917	737.63970	0.137	LOW 1-3, HIGH 1-3	0.355, 1.823
17:0-14:1 PG-d5	Ultimate	Train	[M-H]-	710.50246	3.885	711.50931	-0.603	Calibration Points 1-6	0.067, 0.167, 0.50, 1.0, 2.0, 3.333
17:0-16:1 PG-d5	Ultimate	Train	[M-H]-	738.53382	4.418	739.54061	-0.662	Calibration Points 1-6	0.133, 0.333, 1.0, 2.0, 4.0, 6.667
17:0-18:1 PG-d5	Ultimate	Train	[M-H]-	766.56515	5.110	767.57191	-0.677	Calibration Points 1-6	0.20, 0.50, 1.50, 3.0, 6.0, 10.0
17:0-20:3 PG-d5	Ultimate	Int. Val	[M-H]-	790.56513	4.735	791.57191	-0.631	Calibration Points 1-6	0.133, 0.333, 1.0, 2.0, 4.0, 6.667
17:0-22:4 PG-d5	Ultimate	Train	[M-H]-	816.58052	4.946	817.58761	-0.232	Calibration Points 1-6	0.067, 0.167, 0.50, 1.0, 2.0, 3.333
15:0-18:1(d7) PG	Lipidomix	Ext. Val	[M-H]-	740.54633	4.366	741.55370	0.127	LOW 1-3, HIGH 1-3	0.334, 1.717
17:0-14:1 PC-d5	Ultimate	Train	[M+Acetate-H]-	781.57598	4.645	722.56220	0.098	Calibration Points 1-6	0.133, 0.333, 1.0, 2.0, 4.0, 6.667
17:0-16:1 PC-d5	Ultimate	Train	[M+Acetate-H]-	809.60742	5.387	750.59350	-0.092	Calibration Points 1-6	0.267, 0.667, 2.0, 4.0, 8.0, 13.333
17:0-18:1 PC-d5	Ultimate	Train	[M+Acetate-H]-	837.63872	6.272	778.62480	-0.089	Calibration Points 1-6	0.40, 1.0, 3.0, 6.0, 12.0, 20.0
17:0-20:3 PC-d5	Ultimate	Train	[M+Acetate-H]-	861.63853	5.789	802.62480	0.151	Calibration Points 1-6	0.267, 0.667, 2.0, 4.0, 8.0, 13.333
17:0-22:4 PC-d5	Ultimate	Train	[M+Acetate-H]-	887.65432	6.055	828.64050	0.037	Calibration Points 1-6	0.133, 0.333, 1.0, 2.0, 4.0, 6.667
15:0-18:1(d7) PC	Lipidomix	Ext. Val	[M+Acetate-H]-	811.61989	5.316	752.60610	0.081	LOW 1-3, HIGH 1-3	1.844, 9.481
17:0-14:1 PE-d5	Ultimate	Int. Val	[M-H]-	679.50780	4.774	680.51530	0.329	Calibration Points 1-6	0.067, 0.167, 0.50, 1.0, 2.0, 3.333
17:0-16:1 PE-d5	Ultimate	Int. Val	[M-H]-	707.53926	5.534	708.54660	0.090	Calibration Points 1-6	0.133, 0.333, 1.0, 2.0, 4.0, 6.667
17:0-18:1 PE-d5	Ultimate	Train	[M-H]-	735.57064	6.423	736.57790	-0.022	Calibration Points 1-6	0.20, 0.50, 1.50, 3.0, 6.0, 10.0
17:0-20:3 PE-d5	Ultimate	Tain	[M-H]-	759.57053	5.945	760.57790	0.124	Calibration Points 1-6	0.133, 0.333, 1.0, 2.0, 4.0, 6.667
17:0-22:4 PE-d5	Ultimate	Train	[M-H]-	785.58620	6.201	786.59350	0.031	Calibration Points 1-6	0.067, 0.167, 0.50, 1.0, 2.0, 3.333
15:0-18:1(d7) PE	Lipidomix	Ext. Val	[M-H]-	709.55185	5.465	710.55910	-0.037	LOW 1-3, HIGH 1-3	0.065, 0.336
C16:1 Ceramide-d7 (d18:1-d7/16:1)	Ultimate	Train	[M+Acetate-H]-	601.55405	4.937	542.54040	0.370	Calibration Points 1-6	0.20, 0.50, 1.50, 3.0, 6.0, 10.0
C18:1 Ceramide-d7 (d18:1-d7/18:1)	Ultimate	Train	[M+Acetate-H]-	629.58525	5.783	570.57170	0.528	Calibration Points 1-6	0.133, 0.333, 1.0, 2.0, 4.0, 6.667
C20:1 Ceramide-d7 (d18:1-d7/20:1)	Ultimate	Train	[M+Acetate-H]-	657.61669	6.649	598.60300	0.269	Calibration Points 1-6	0.067, 0.167, 0.50, 1.0, 2.0, 3.333
C22:1 Ceramide-d7 (d18:1-d7/22:1)	Ultimate	Int. Val	[M+Acetate-H]-	685.64793	7.166	626.63430	0.353	Calibration Points 1-6	0.133, 0.333, 1.0, 2.0, 4.0, 6.667
C24:1 Ceramide-d7 (d18:1-d7/24:1)	Ultimate	Train	[M+Acetate-H]-	713.67927	7.461	654.66560	0.276	Calibration Points 1-6	0.20, 0.50, 1.50, 3.0, 6.0, 10.0
17:0-14:1 DG-d5	Ultimate	Train	[M+Acetate-H]-	616.52061	6.104	557.50680	0.074	Calibration Points 1-6	0.067, 0.167, 0.50, 1.0, 2.0, 3.333
17:0-16:1 DG-d5	Ultimate	Train	[M+Acetate-H]-	644.55181	6.914	585.53810	0.241	Calibration Points 1-6	0.133, 0.333, 1.0, 2.0, 4.0, 6.667
17:0-18:1 DG-d5	Ultimate	Train	[M+Acetate-H]-	672.58312	7.287	613.56940	0.214	Calibration Points 1-6	0.20, 0.50, 1.50, 3.0, 6.0, 10.0
17:0-20:3 DG-d5	Ultimate	Train	[M+Acetate-H]-	696.58315	7.113	637.56940	0.158	Calibration Points 1-6	0.133, 0.333, 1.0, 2.0, 4.0, 6.667
17:0-22:4 DG-d5	Ultimate	Int. Val	[M+Acetate-H]-	722.59874	7.197	663.58500	0.167	Calibration Points 1-6	0.067, 0.167, 0.50, 1.0, 2.0, 3.333
15:0-18:1(d7) DG	Lipidomix	Ext. Val	[M+Acetate-H]-	646.56447	6.872	587.55060	-0.032	LOW 1-3, HIGH 1-3	0.108, 0.555

Table S4b. List of all lipid standards and the corresponding dataset (Train/Internal Validation/External Validation), primary observed ion adduct, the corresponding *m/z*, retention time, monoisotopic mass, mass error, samples containing that specific standard, and the spike concentration for standards in negative ion mode.

(a)

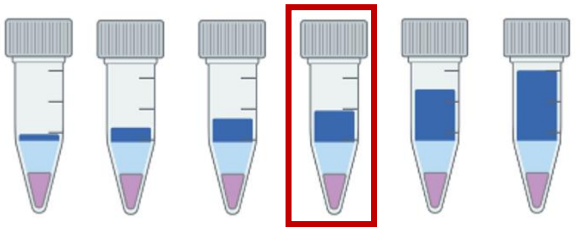

Avanti Splash Lipidomix (14 unique deuterated lipids)
Avanti Ultimate SplashOne mix (69 unique deuterated lipids)

$$x_{Lipidomix} = x_{Ultimate}\,(y_{Lipidomix} / y_{Ultimate})$$

	UltimateSplash	Splash Lipidomix
**Analyte**	16:0-17:1-16:0 TG-d5	15:0-18:1(d7)-15:0 TG
Response	1,968,108,490	173,471,530
Concentration	125ug/mL	Treated as Unknown

x = 125[(173,471,530)/(1,968,108,490)] = 11.02

Calculated Concentration of 15:0-18:1(d7)-15:0 TG in this sample would be 11.0 ug/mL.

(b)

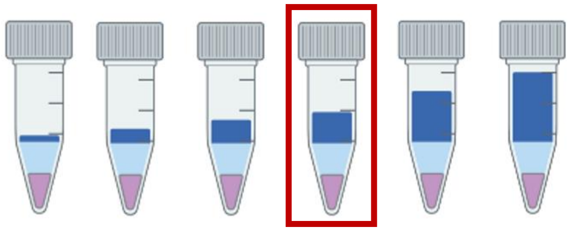

Avanti Splash Lipidomix (14 unique deuterated lipids)
Avanti Ultimate SplashOne mix (69 unique deuterated lipids)

$$x_{Lipidomix} = y_{Lipidomix} / m_{Ultimate\ (Class)}$$

Ultimate SplashOne TG's

14:0-13:0-14:0 TG-d5	25 µg/mL
14:0-15:1-14:0 TG-d5	50 µg/mL
14:0-17:1-14:0 TG-d5	75 µg/mL
16:0-15:1-16:0 TG-d5	100 µg/mL
16:0-17:1-16:0 TG-d5	125 µg/mL
16:0-19:2-16:0 TG-d5	100 µg/mL
18:1-17:1-18:1 TG-d5	75 µg/mL
18:1-19:2-18:1 TG-d5	50 µg/mL
18:1-21:2-18:1 TG-d5	25 µg/mL

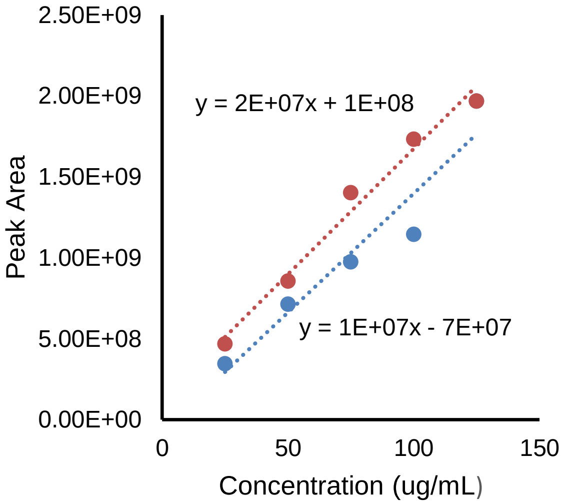

The average of the two slopes is 1.5E+07
x = (173,471,530)/(15,000,000) = 11.56

Calculated Concentration of 15:0-18:1(d7)-15:0 TG in this sample would be 11.6 ug/mL.

Figure S1. Example calculations for other semi-quantitative approaches. (a) One-point calibration, and (b) surrogate multi-point calibration. Note that, in this example, the concentrations in the original stock vial were calculated, not the spiked concentrations. However, application of the dilution factor would yield the spiked concentrations.

**Splash Lipidomix Standard**	**Ultimate Splash Standard Used for One-Point Quantitation**
18:1(d7) Lyso PE	17:0 Lyso PE-d5
18:1(d7) Lyso PC	17:0 Lyso PC-d5
(15:0/18:1-d7) PI	17:0-18:1 PI-d5
18:1 SM (d18:1/18:1)-d9	20:1 SM (d18:1/20:1)-d9
(15:0/18:1-d7) PG	17:0-18:1 PG-d5
(15:0/18:1-d7) PC	17:0-18:1 PC-d5
(15:0/18:1-d7) PE	17:0-18:1 PE-d5
(15:0/18:1-d7) DG	17:0-18:1 DG-d5
15:0/18:1/15:0 TG-d7	16:0-17:1-16:0 TG-d5

Table S5. List of corresponding Ultimate Splash standards used for quantitation of Splash Lipidomix standards in one-point calibration.

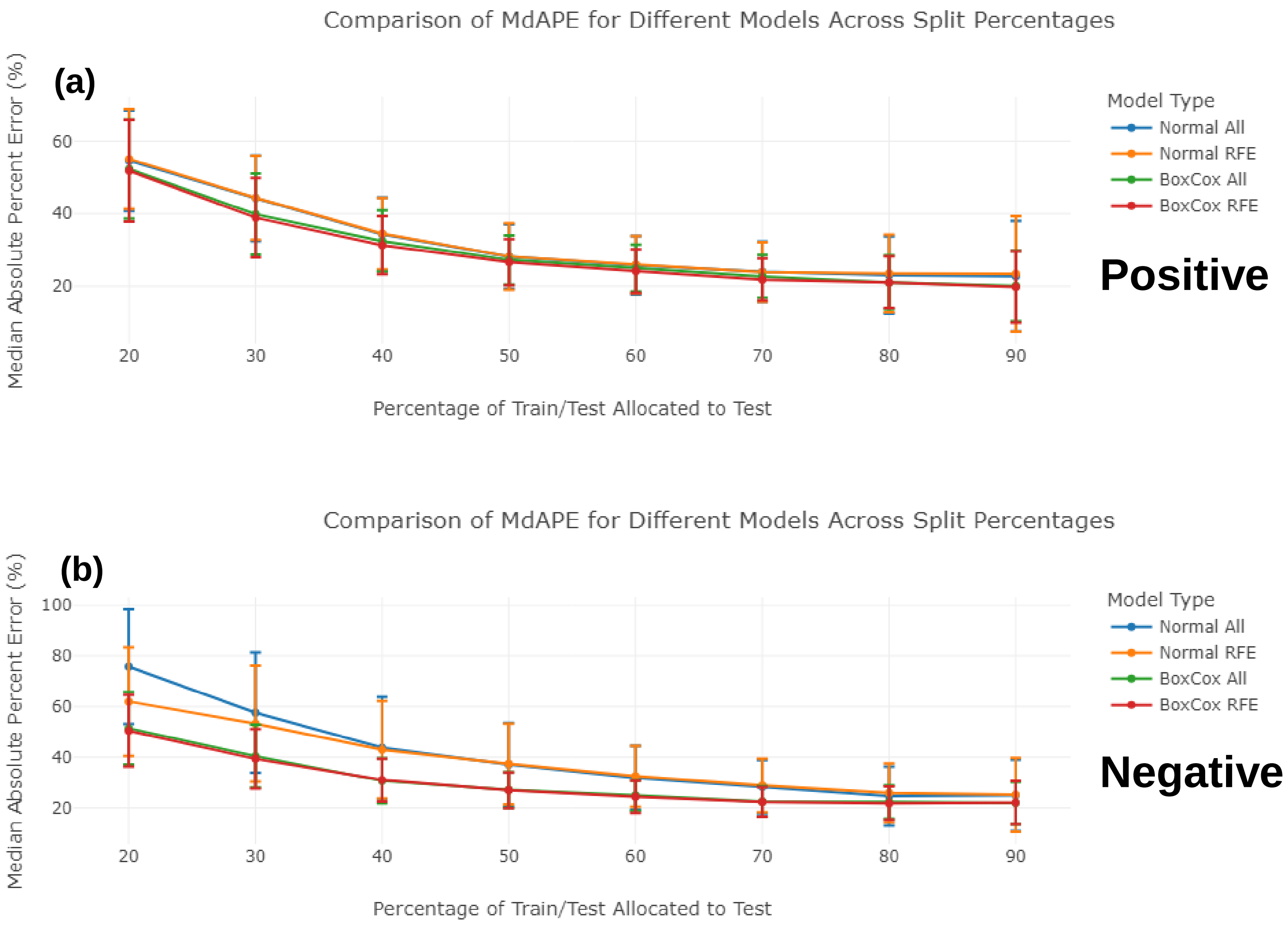

Figure S2. Learning curves for (a) positive and (b) negative ion mode models across different train/test split percentages. Both learning curves were created with the median absolute percent error (MdAPE) in prediction of lipid sensitivities (m_R) of UltimateSplash components in Donor 1 samples. The “Normal All” blue trace is for prediction of m_R without transformation and without feature selection. The “Normal RFE” orange trace is for the prediction of m_R with recursive feature elimination and no transformation. “BoxCox All” green trace is prediction of m_R with Box-Cox transformation and no feature selection, and finally the red trace “BoxCox RFE” is prediction of m_R with transformation and recursive feature elimination.

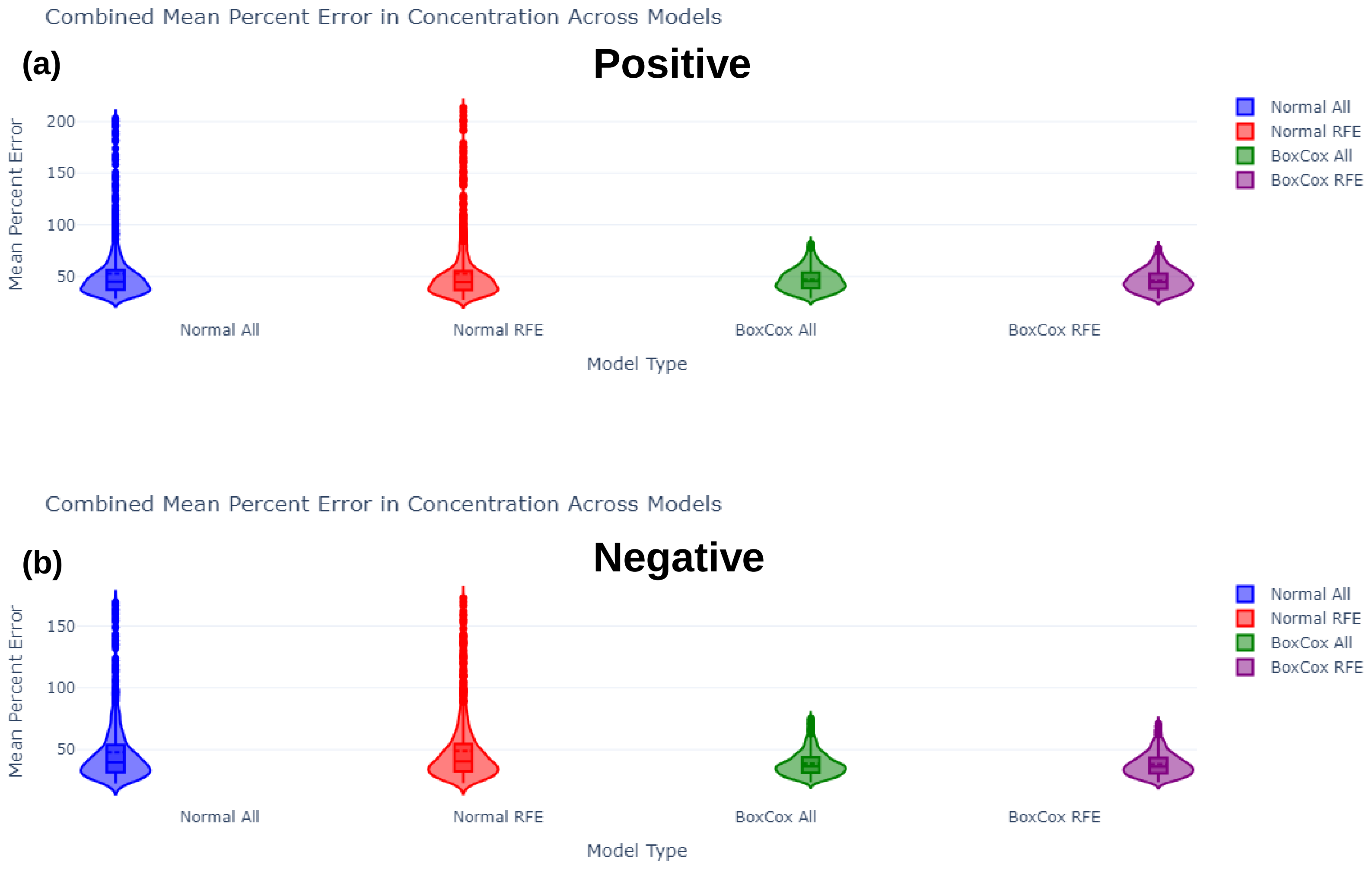

Figure S3. Assessment of model performance for various approaches to data transformation and variable selection. Violin plots of (a) mean percent error in m_R prediction for different positive mode model types and (b) mean percent error in prediction of m_R for different negative mode model types. Prediction errors were calculated for the UltimateSplash components in Donor 1 samples.

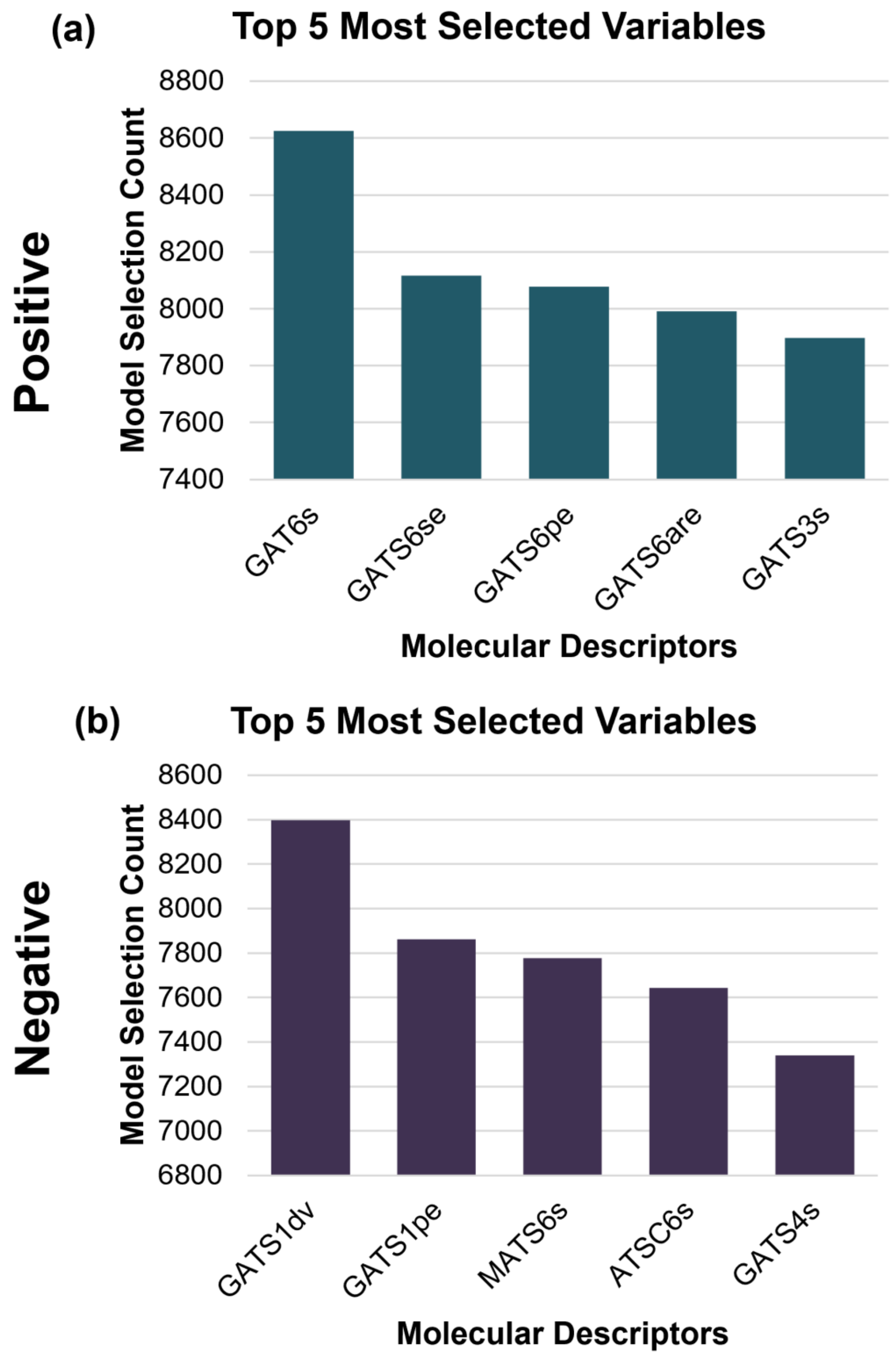

Figure S4. Commonly selected variables in model iterations. Top 5 most selected variables in the 1101 model iterations performed for (a) positive mode and (b) negative mode.

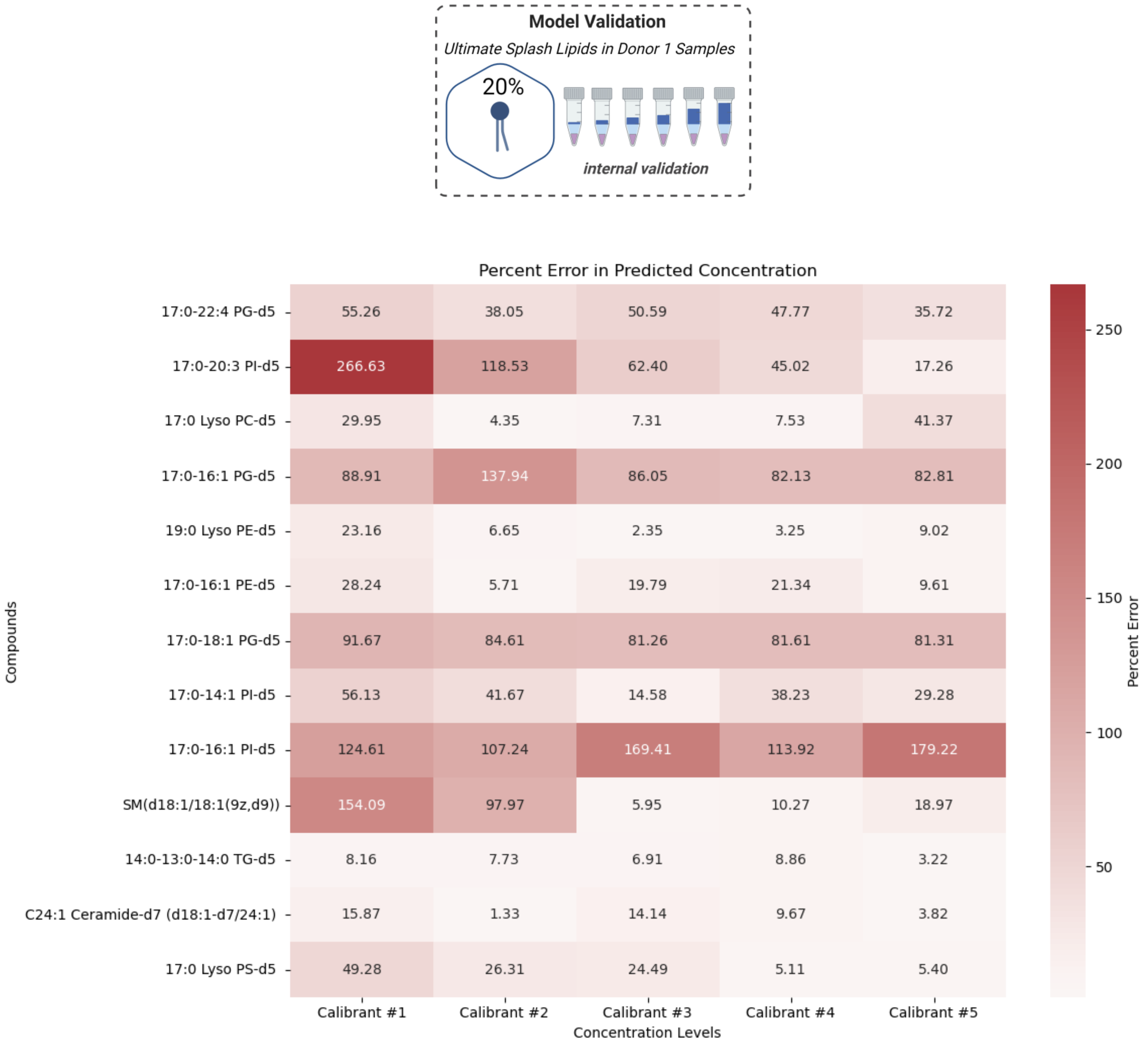

Figure S5. Positive ion mode internal validation heatmap of predicted concentration percent errors by sample. Predictions were made for the 20% percent withheld UltimateSplash lipids in Donor 1 samples. Calibrant concentration levels correspond to those in the rightmost column of Supplemental Table S3a. The highest calibrant level (#6) was removed in all cases, as it was well outside of the linear response range.

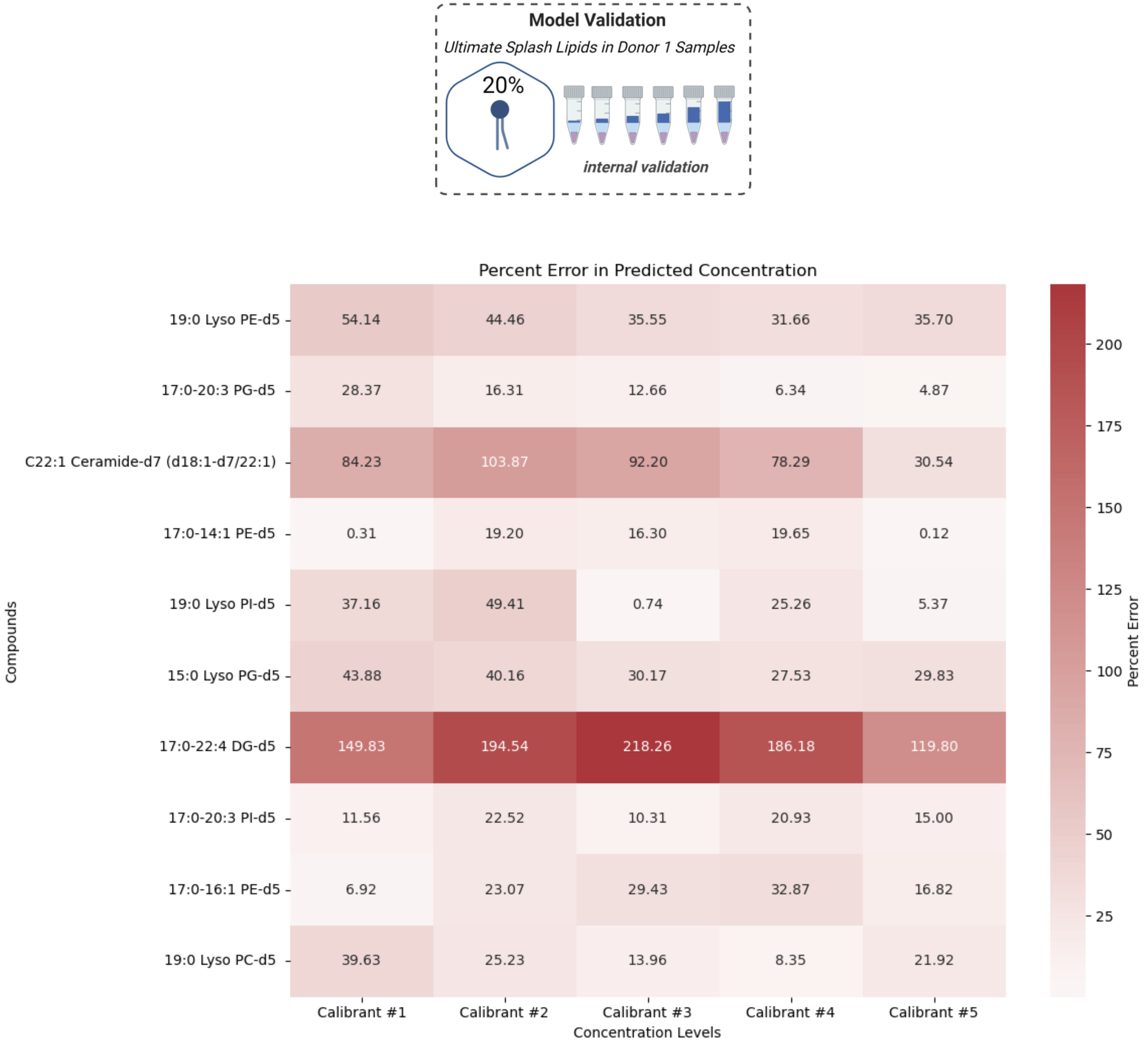

Figure S6. Negative ion mode internal validation heatmap of predicted concentration percent errors by sample. Predictions were made for the 20% percent withheld UltimateSplash lipids in Donor 1 samples. Calibrant concentration levels correspond to those in the rightmost column of Supplemental Table S3b. The highest calibrant level (#6) was removed in all cases, as it was well outside of the linear response range.

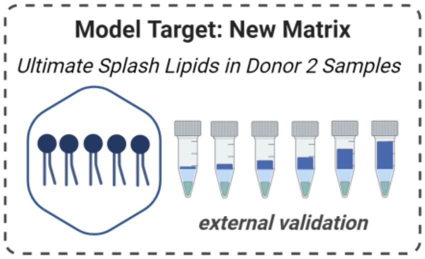

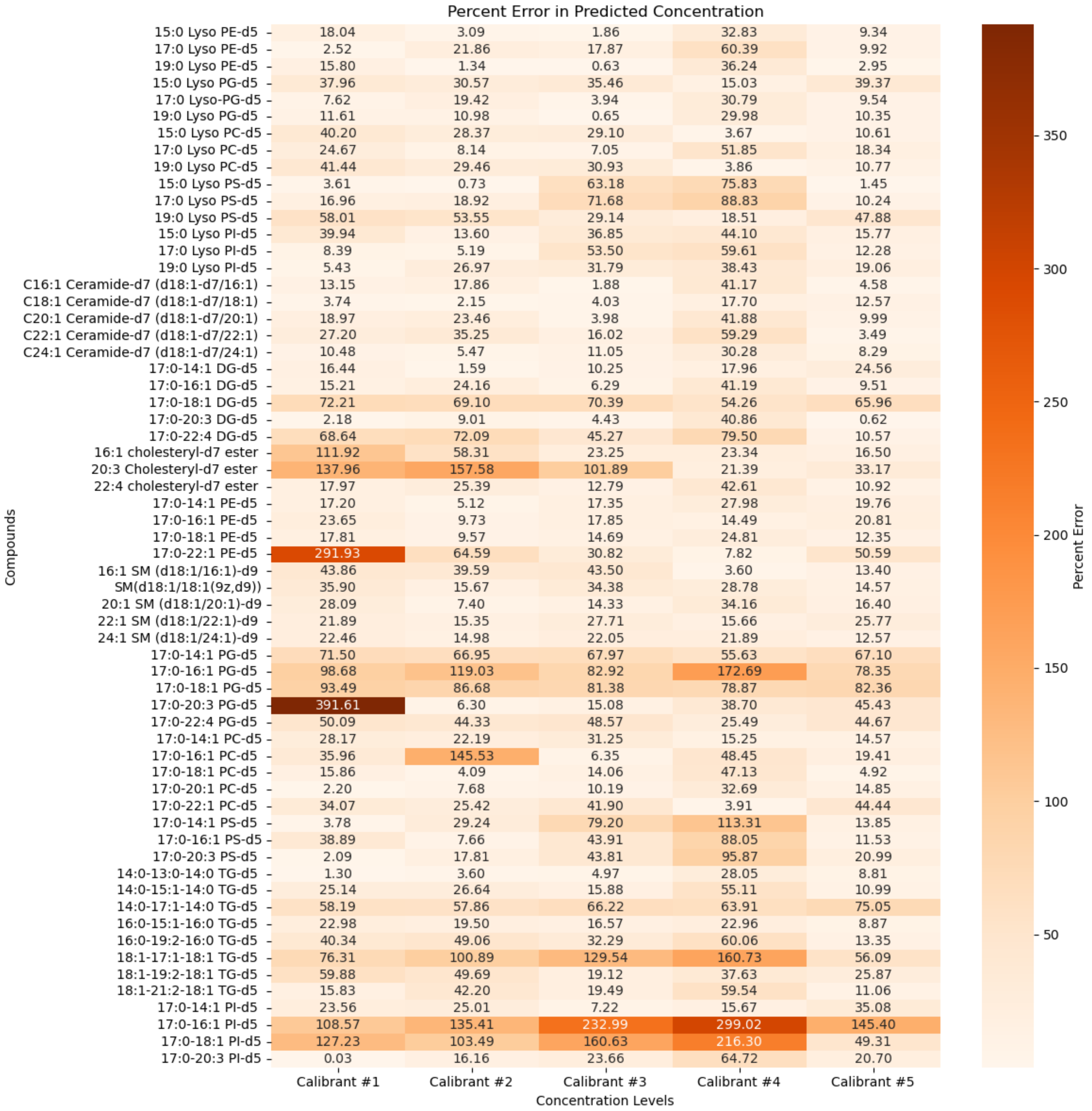

Figure S7. Positive mode external validation heatmap of predicted concentration percent errors by sample. Predictions were made for Ultimate Splash lipids in Donor 2 samples.

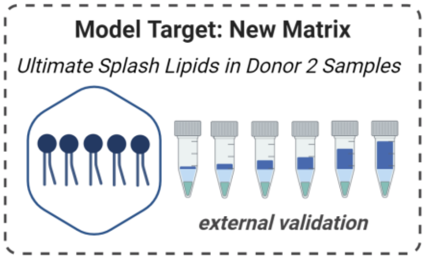

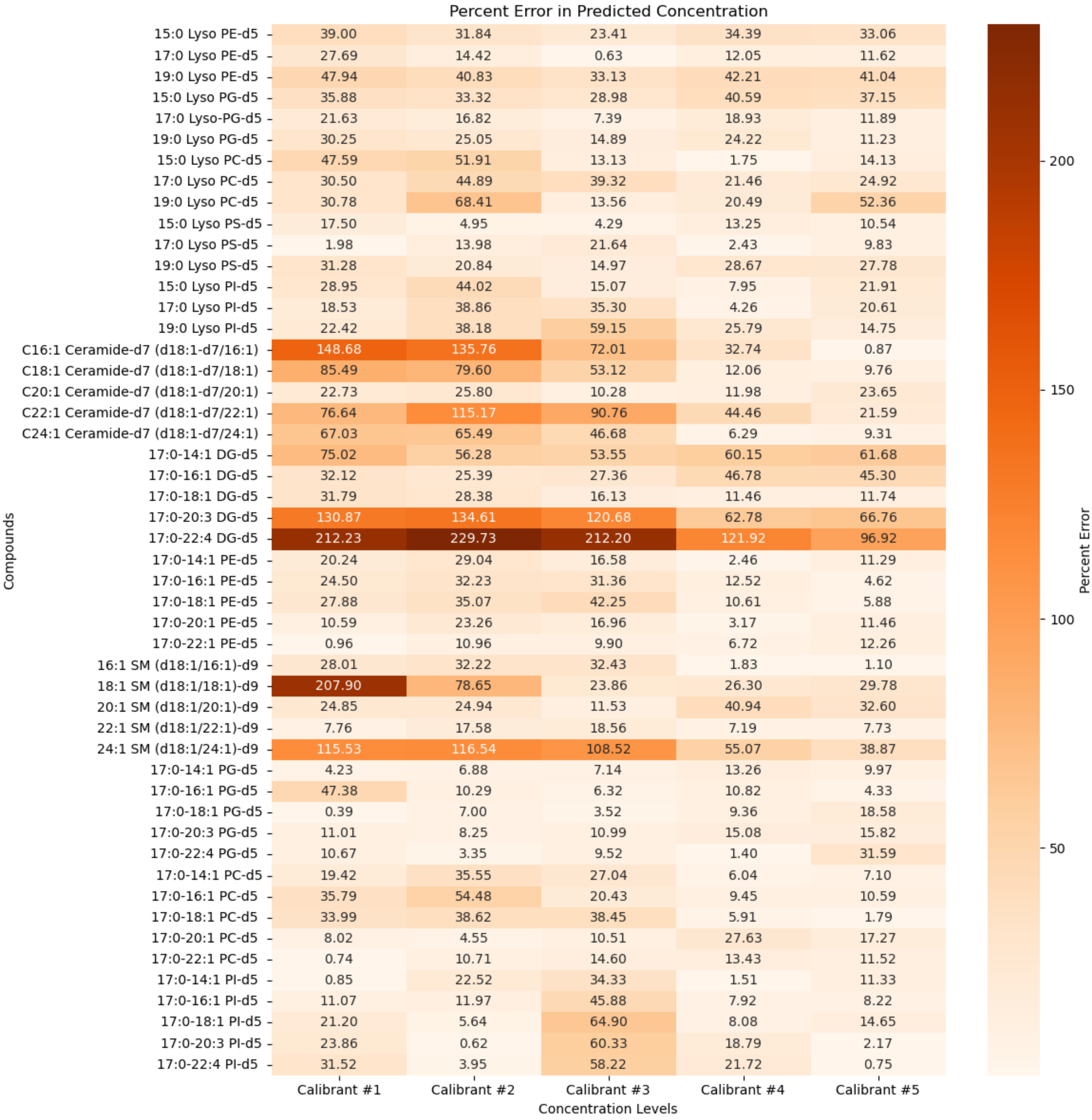

Figure S8. Negative mode external validation heatmap of predicted concentration percent errors by sample. Predictions were made for Ultimate Splash lipids in Donor 2 samples.

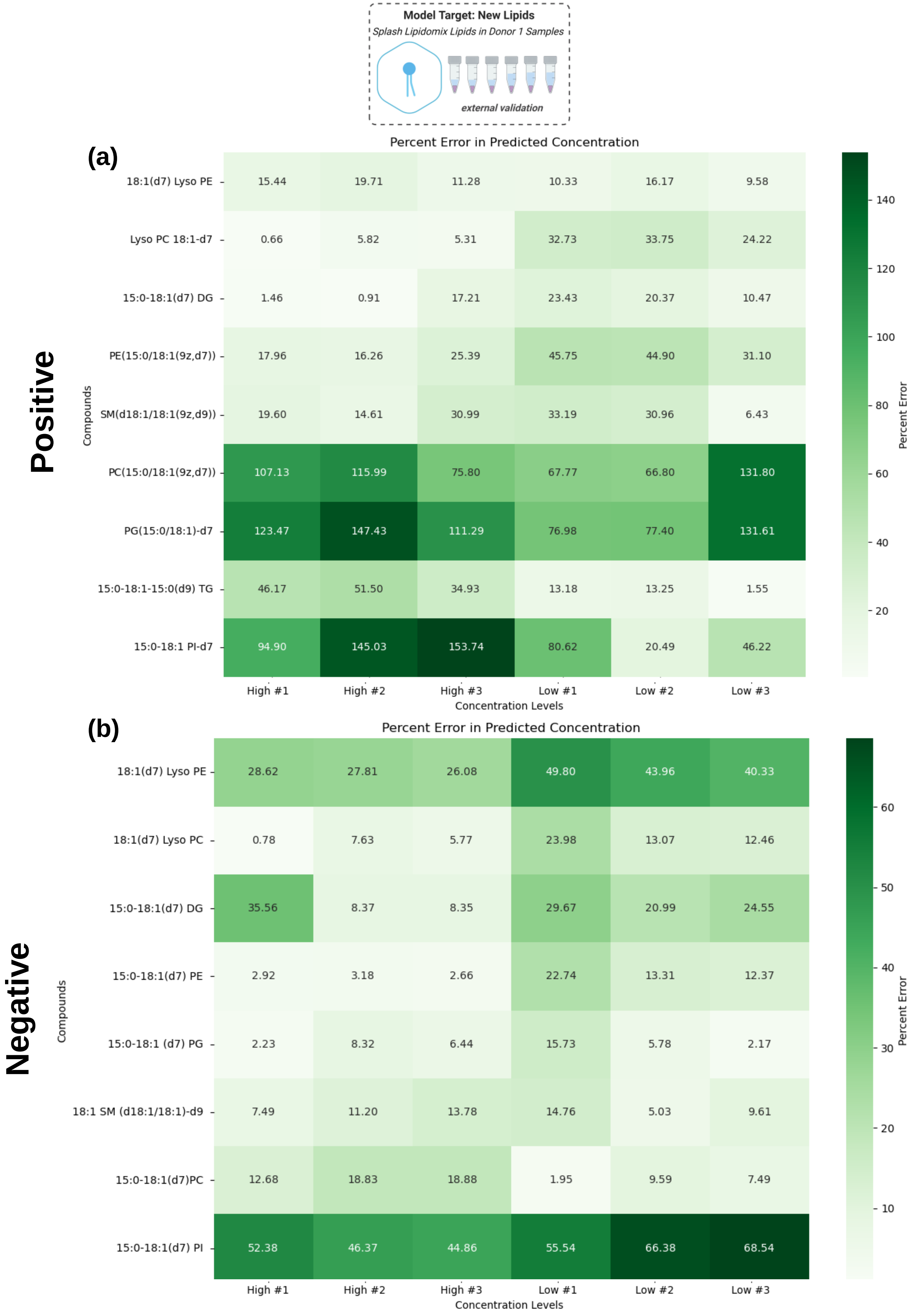

Figure S9. External validation heatmap of predicted concentration percent errors by sample in a) positive mode and b) negative mode. Predictions were made for Splash Lipidomix lipids in Donor 1 samples.

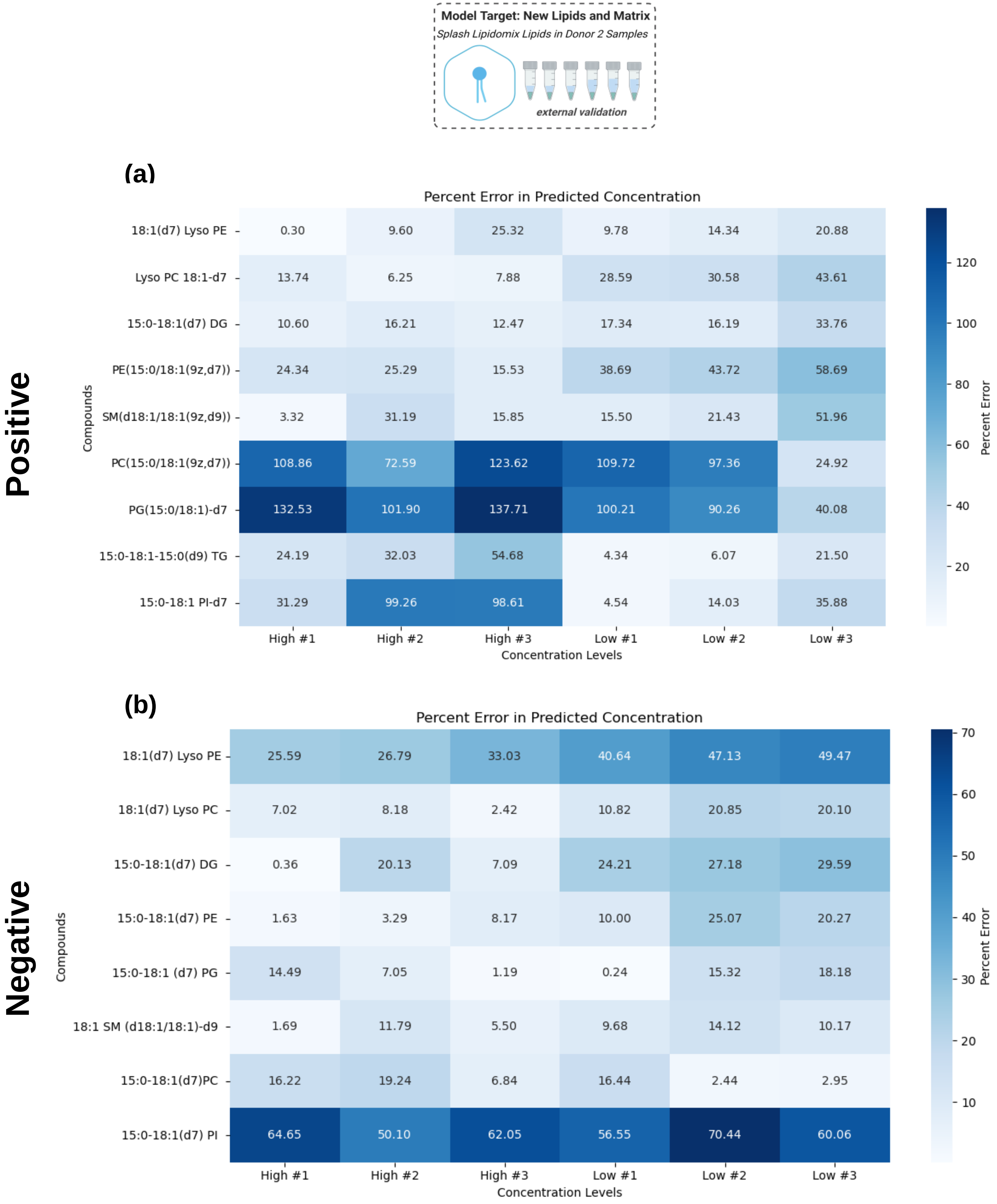

Figure S10. External validation heatmap of predicted concentration percent errors by sample in a) positive mode and b) negative mode. Predictions were made for Splash Lipidomix lipids in Donor 2 samples.

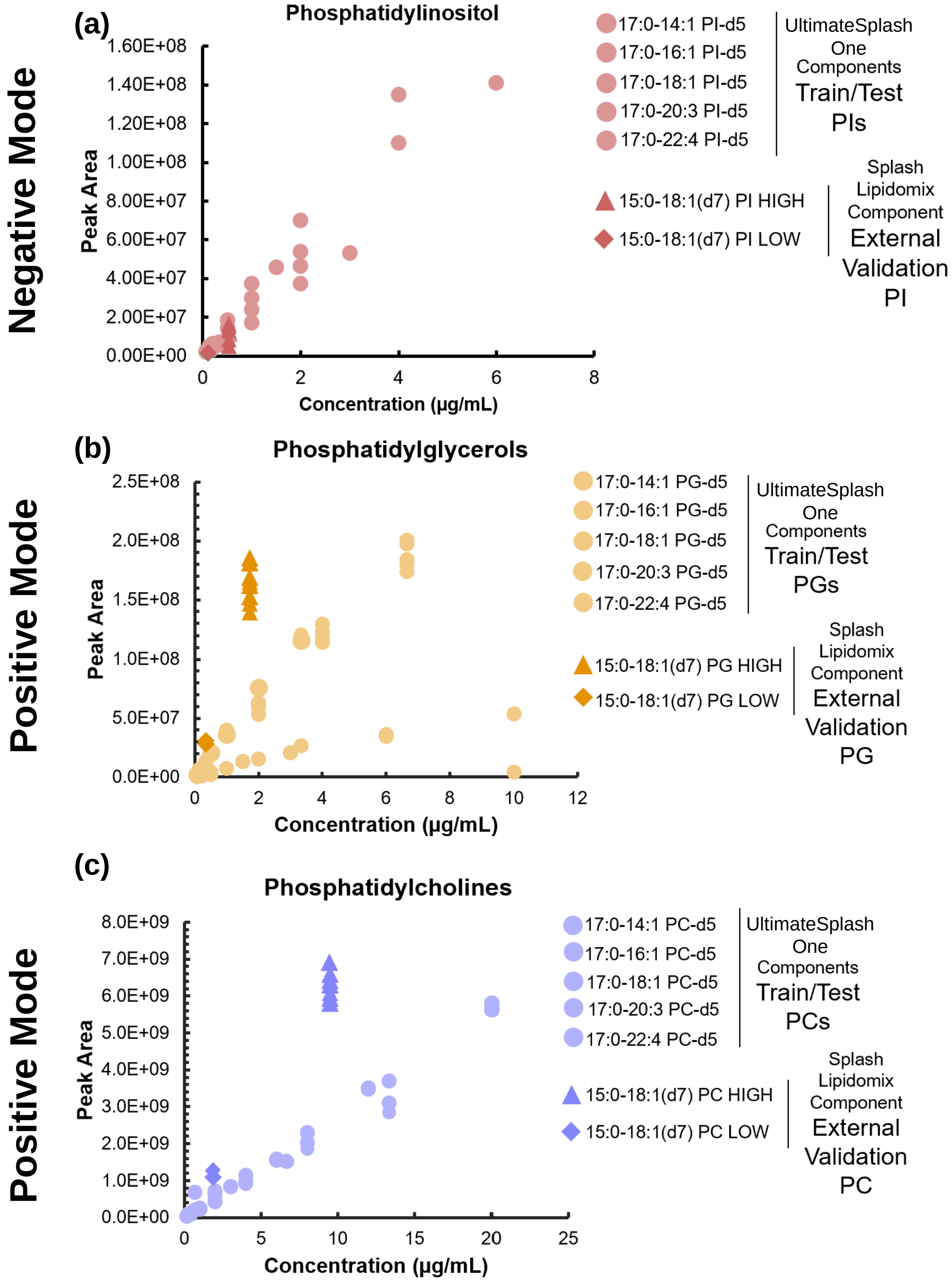

Figure S11. Outlier Analysis. Peak area – concentration plots for (a) PI standards in negative ion mode, (b) PG standards in positive ion mode, and c) PC standards in positive ion mode. All plots depict responses for Donor 1 samples.

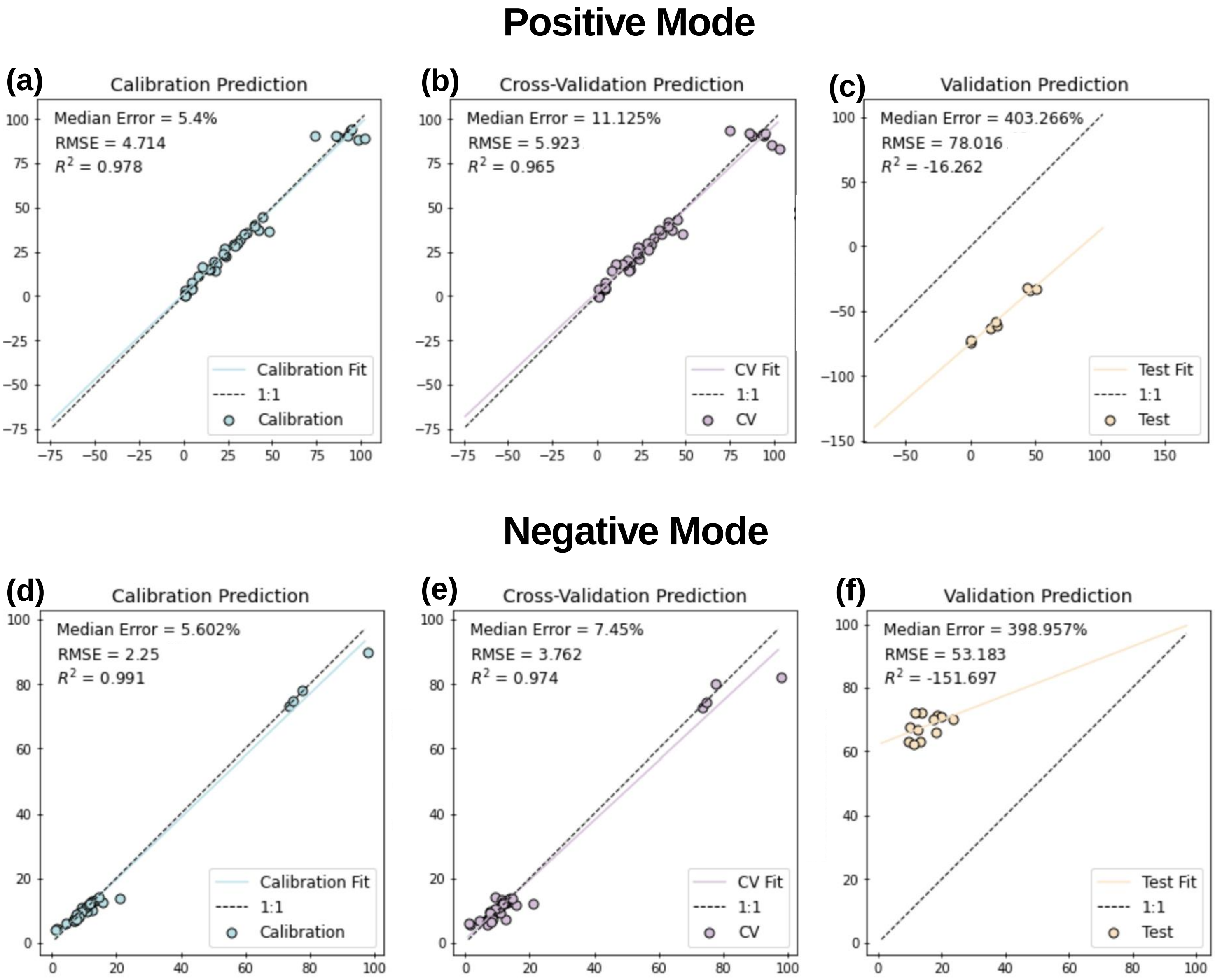

Figure S12. Model Performance on Unseen Lipid Classes. Models run in positive mode (a-c) and negative mode (d-f) where all non-Lyso lipids in the Ultimate Splash mix were used to train the model for calibration (a,d), and all Lyso lipids in the Ultimate Spash mix were contained in the validation set (c,f). X-axes are experimental ionization efficiency measurements, m_R, used as ground truth. These experiments were performed with independent Box-Cox optimization so experimental values are different from those presented in previous sections of the manuscript. Y-axes are the predicted m_R values.

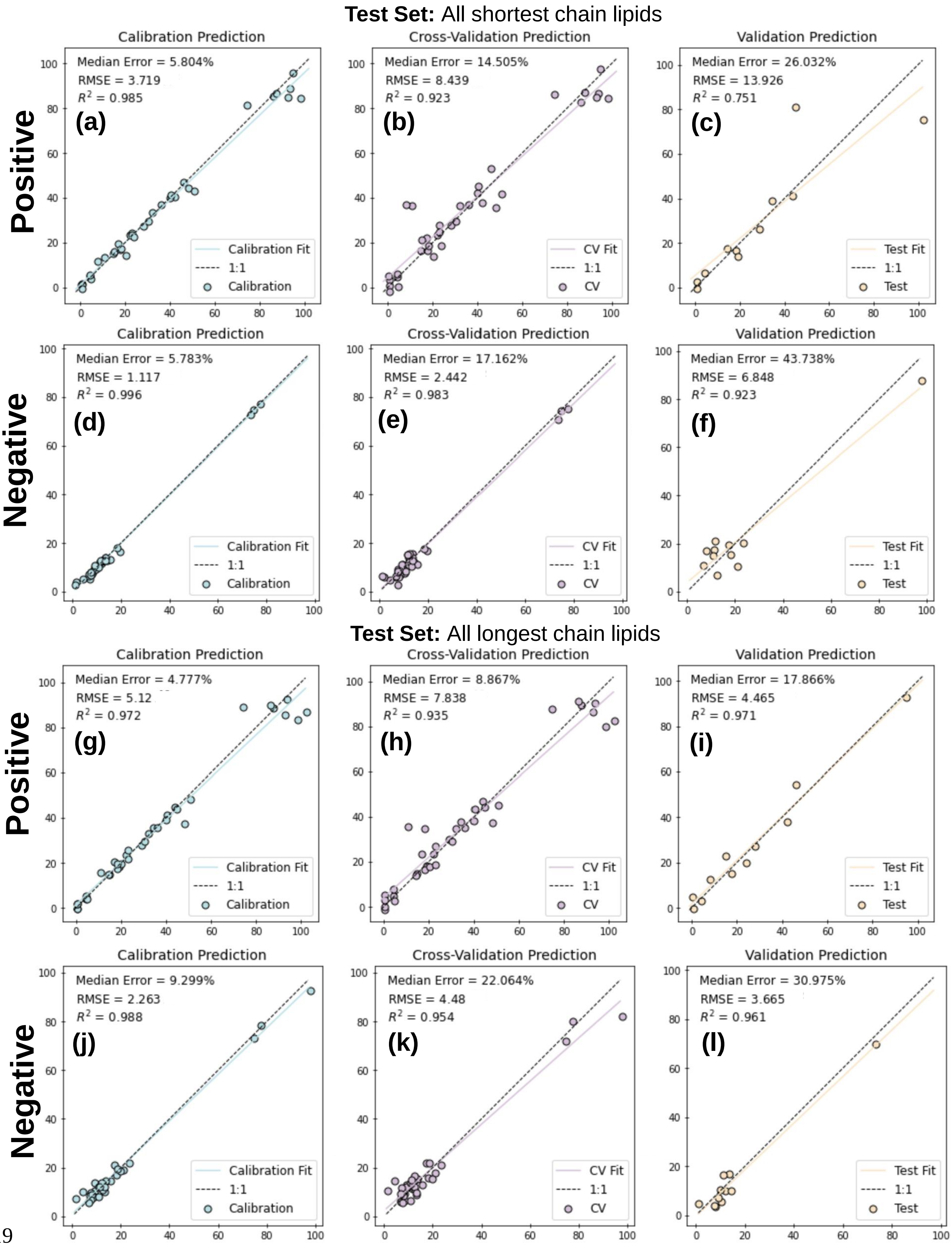

Figure S13. Model Performance on Unseen Lipid Masses. Models run in positive mode (a-c) and negative mode (d-f) where the smallest (by mass) lipids from each class in the UltimateSplash mix were designated in a test set for validation (c,f) while calibration is performed using the training set composed of all remaining lipids in the mix (a,d). Additionally, models run in positive mode (g-i) and negative mode (j-l) where the largest (by mass) lipids from each class in the UltimateSplash mix were designated in a test set for validation (i,l) while calibration is performed using the training set composed of all remaining lipids in the mix (g,j). X-axes are experimental ionization efficiency measurements, m_R, used as ground truth. These experiments were performed with independent Box-Cox optimization so experimental values are different from those presented in previous sections of the manuscript. Y-axes are the predicted m_R values.
